# Efficient access to 3′-*O*-phosphoramidite derivatives of tRNA related *N*^6^-threonylcarbamoyladenosine (t^6^A) and 2-methylthio-*N*^6^-threonylcarbamoyladenosine (ms^2^t^6^A)†

**DOI:** 10.1039/d0ra09803e

**Published:** 2021-01-07

**Authors:** Katarzyna Debiec, Elzbieta Sochacka

**Affiliations:** Institute of Organic Chemistry, Lodz University of Technology Zeromskiego 116 90-924 Lodz Poland elzbieta.sochacka@p.lodz.pl

## Abstract

An efficient method of ureido linkage formation during epimerization-free one-pot synthesis of protected hypermodified *N*^6^-threonylcarbamoyladenosine (t^6^A) and its 2-SMe analog (ms^2^t^6^A) was developed. The method is based on a Tf_2_O-mediated direct conversion of the *N*-Boc-protecting group of *N*-Boc-threonine into the isocyanate derivative, followed by reaction with the *N*^6^*exo*-amine function of the sugar protected nucleoside (yield 86–94%). Starting from 2′,3′,5′-tri-*O*-acetyl protected adenosine or 2-methylthioadenosine, the corresponding 3′-*O*-phosphoramidite monomers were obtained in 48% and 42% overall yield (5 step synthesis). In an analogous synthesis, using the 2′-*O*-(*tert*-butyldimethylsilyl)-3′,5′-*O*-(di-*tert*-butylsilylene) protection system at the adenosine ribose moiety, the t^6^A-phosphoramidite monomer was obtained in a less laborious manner and in a remarkably better yield of 74%.

## Abstract 2

A new convenient method of ureido linkage formation in t^6^A/ms^2^t^6^A nucleosides greatly enhances the availability of phosphoramidite monomers for the chemical synthesis of modified tRNA fragments.

## Graphical abstract

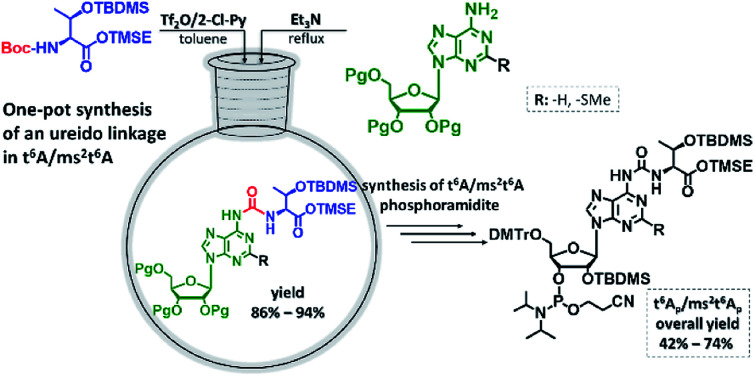

## Introduction

Transfer RNAs (tRNAs) are known for having a substantial content of modified nucleoside units.^1,2^ To date, in tRNAs from all domains of life, more than 130 modified units have been identified, which differ in chemical structure,^3–6^ distribution within the tRNA molecules,^7^ and their biological activity.^8–13^

**Fig. 1:**
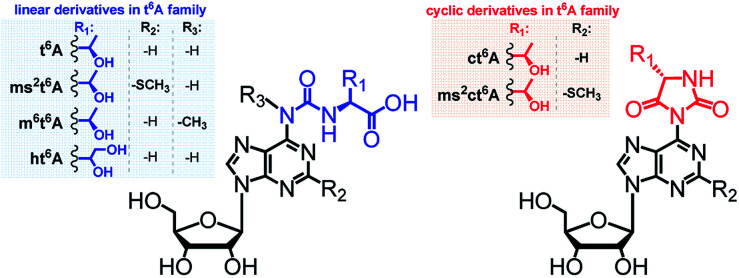
Abbreviations and structures of l-threonylcarbamoyl modified adenosines (the t^6^A_37_ family) located in tRNAs at the position 37.

The majority of modified units are present in the anticodon loop and stem domain of tRNAs, particularly at position 34 (the wobble position) and at position 37, *i.e.* adjacent to the anticodon at its 3′-side.^3,7,14,15^ Considering the latter modifications, special interest has been paid to several *N*^6^-threonylcarbamoyladenosines (depicted in Fig. 1), which are widely involved in the decoding of the A-starting codons (ANN).^15,16^ Among them, the most abundant *N*^6^-threonylcarbamoyladenosine (t^6^A)^17^ and its analogs containing either the –SMe group at the purine C2 atom (ms^2^t^6^A),^18^ or the methyl substituent at the *N*^6^-atom (m^6^t^6^A)^19^ have been known for many years and their diverse functions during the protein biosynthesis were intensively studied.^6,9,15,16^ Recently, next members of the t^6^A family have been identified in the tRNA anticodon loops, *i.e.* cyclic *N*^6^-threonylcarbamoyladenosine (ct^6^A)^20,21^ cyclic 2-methylthio-*N*^6^-threonylcarbamoyladenosine (ms^2^ct^6^A),^22^ and a t^6^A derivative having the threonine methyl group converted into a hydroxymetyl one (hydroxy-*N*^6^-threonylcarbamoyl-adenosine, ht^6^A).^23^

**Scheme 1:**
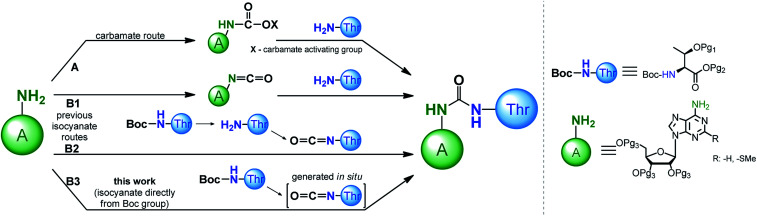
Approaches for the formation of the ureido linkage in t^6^A modified nucleoside.

Recognition of the structural aspects and biological functions of the t^6^A nucleoside family is highly dependent on the synthetic availability of these nucleosides, as well as their 3′-*O*-phosphoramidite derivatives, which are essential for fast and efficient synthesis of model oligonucleotides with the sequence of the appropriate tRNA anticodon stems and loops (ASL of tRNAs). To date, several procedures have been developed to modify adenosine or 2-methylthioadenosine (ms^2^A) at the *N*^6^ position with a threonylcarbamoyl chain (a ureido system is formed) using either a carbamate or isocyanate approach (Scheme 1, paths A and B, respectively).

Adenosine *N*^6^-ethyl carbamate was used in the first syntheses of “free” t^6^A nucleoside,^24–27^ as well as in the preparation of its stereoisomers containing l, d, l-*allo*, and d-*allo*-Thr.^28^ Analogously, t^6^A derivatives suitable for synthesis of the corresponding 3′-*O*-phosphoramidite derivative (protected with *tert*-butyldimethylsilyl (TBDMS) on the OH and trimethylsilylethyl ester (TMSE) at the COOH of threonine residue) can be prepared.^29^ The carbamate method has been significantly improved by the use of more active phenyl carbamate derivatives of adenosine/2-methylthio-adenosine.^30–40^ This was possible with phenoxycarbonyl tetrazole^31^ or 1-*N*-methyl-3-phenoxycarbonyl-imidazolium chloride^41^ used as effective reagents introducing the carbamate functionality onto the weakly nucleophilic *N*^6^-amine function of A/ms^2^A nucleosides.

The isocyanate approach to the synthesis of t^6^A/ms^2^t^6^A (Scheme 1, path B1,2) was shown to have limited applicability in the preparation of “free” nucleosides.^24^ Because this method required a threonine derivative protected on the OH and COOH functions, it was considered inferior to the carbamate approach in which unprotected amino acid can be used.^24–26^ However, the isocyanate route was recently postulated by the Carell's group as a possible pathway for the formation of t^6^A under prebiotic conditions.^42^

In the synthesis of threonine protected t^6^A/ms^2^t^6^A derivatives for the subsequent preparation of the corresponding 3′-*O*-phosphoramidites, the isocyanate approach^39,43^ is much less explored than the carbamate procedures.^29,34–40^ Initially, the isocyanate derivative was generated from the *N*^6^-amine function of sugar protected adenosine (Scheme 1, path B1), but its condensation with the free amine function of l-threonine was ineffective and the ureido-nucleoside product was obtained in a low 19% yield.^43^ Noticeably better results were obtained in our recently published method (Scheme 1, path B2), based on the reaction of isocyanate derivative of the amino acid substrate (prepared by removing of Boc-protection and phosgene treatment of the free amine function of l-threonine appropriately blocked on the OH and COOH functions) with the sugar protected nucleoside (overall yield of this three steps procedure ∼55%).^39^ This result of isocyanate procedure turned our attention to the methods of synthesis of unsymmetrical ureas involving the formation of the isocyanate functionality directly from the carbamate type protecting groups of amino acids (*e.g. N*-Boc protecting group).^44–53^ Most likely, such variant of the isocyanate method (Scheme 1, path B3) applied in the synthesis of the 3′-*O*-phosphoramidite derivatives of t^6^A/ms^2^t^6^A would be greatly advantageous in comparison to our previous isocyanate route (Scheme 1, path B2) owing to a smaller number of synthesis steps in the preparation of threonine derivative (the removal of *N*-Boc protection is unnecessary) and escaping the use of toxic phosgene.

Here we report a new one-pot procedure for the introduction of an ureido linkage into t^6^A/ms^2^t^6^A using a Tf_2_O-mediated generation of the isocyanate derivative directly from *N*-Boc-protecting group of l-threonine, followed by its straight reaction with the *N*^6^*exo*-amine function of the sugar protected nucleoside. We have also showed that this approach is compatible with the use of the recently introduced 2′-*O*-(*tert*-butyldimethylsilyl)-3′,5′-*O*-(di-*tert*-butylsilylene) ribonucleoside sugar protection system,^54–56^ that allows to prepare the 3′-*O*-phosphoramidite monomeric unit more effectively and in a less laborious manner.

## Results and discussion

Search for the best conditions leading to the formation of the ureido compound 4a was performed using trimethylsilylethyl (TMSE) ester of *N*-Boc-*O-tert*-butyldimethylsilyl (TBDMS) protected l-threonine^39^ (1) and 2′,3′,5′-tri-*O*-acetyladenosine (3a) (Table 1, see ESI for spectroscopic data of 1, 2, 3a, Fig. S1–S7†). In all cases, the final condensation of isocyanate 2 with 3a was performed in the presence of Et_3_N (2-fold molar excess over the Tf_2_O activator used for isocyanate formation) in boiling toluene for 16 h. It was reported that addition of Et_3_N, which is an effective scavenger of trifluoromethanesulfonic acid (generated in the step of isocyanate formation), helps to maintain a concentration of the unprotonated amine component sufficient for effective nucleophilic attack on the isocyanate moiety.^48,50^

**Table 1:** Optimization of the reaction conditions for the synthesis of t^6^A from Boc-Thr derivative 1 and the sugar-protected adenosine 3a^a^

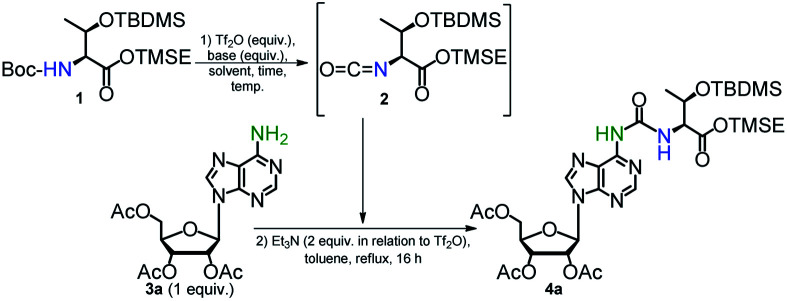				
Entry	Boc-Thr 1^b^ (equiv.)	Tf_2_O/base^c^ (equiv.)	Solvent, time, temp. (°C)	Yield of 4a^d^ (%)
1	1.0	Tf_2_O (1.5)/2-Cl-Py (3.0)	CH_2_Cl_2_, rt, 15 min	19%
2	1.0	Tf_2_O (1.5)/2-Cl-Py (3.0)	CH_2_Cl_2_, 0 °C, 5 min	46%
3	1.0	Tf_2_O (2.0)/2-Cl-Py (4.0)	CH_2_Cl_2_, 0 °C, 5 min	42%
4	1.0	Tf_2_O (1.5)/Py (3.0)	CH_2_Cl_2_, rt^e^, 3 h	—
5	1.0	Tf_2_O (1.5)/DMAP (3.0)	CH_2_Cl_2_, rt^e^, 3 h	—
6	1.0	Tf_2_O (1.5)/Et_3_N (3.0)	CH_2_Cl_2_, rt^e^, 3 h	—
7	1.0	Tf_2_O (1.5)/2,6-lutidine (3.0)	CH_2_Cl_2_, rt^e^, 30 min	16%
8	1.5	Tf_2_O (2.25)/2-Cl-Py (4.5)	CH_2_Cl_2_, 0 °C, 5 min	71%
9	2.0	Tf_2_O (3.0)/2-Cl-Py (6.0)	CH_2_Cl_2_, 0 °C, 5 min	80%
10	2.5	Tf_2_O (3.75)/2-Cl-Py (7.5)	CH_2_Cl_2_, 0 °C, 5 min	92%
11	2.5	Tf_2_O (3.75)/2-Cl-Py (7.5)	Toluene, rt, 15 min	92%

a All reactions were performed in a 0.2 mmol scale in 6 mL of the corresponding solvent.

b The number of equivalents was calculated in respect to the nucleoside reagent 3a.

c The ratios of 1/Tf_2_O = 1.5 and Tf_2_O/base = 2 were applied.

d Isolated yield after column chromatography.

e The reaction was carried out also in 0 °C and after stirring for 3 h no consumption of 1 was observed according to TLC analysis.

To optimize the triflic anhydride (Tf_2_O) mediated conversion of *N*-Boc-protected threonine 1 (a dichloromethane solution) into the isocyanate derivative 2 (the first step of the one-pot synthesis of 4a) we were changing the amount of Tf_2_O activator, basicity of amine, temperature and reaction time (entries 1–7). When the amount of 2 reached the plateau (TLC monitoring) the reaction mixture was concentrated, the residue was dissolved in toluene and Et_3_N and the nucleoside substrate 3a was added. The reaction 1 → 2 for 15 min at room temp. (entry 1), followed by reaction with 3a, afforded the final product 4a in a low 19% yield and several by-products were detected by TLC analysis. An experiment conducted at lower temperature (0 °C) for much shorter time (5 min) (entry 2) was more productive (46% yield) but the yield did not further increase when higher concentration of Tf_2_O (2 equiv.) was used (entry 3). Compound 1 did not react when more common bases such as pyridine, 4-dimethylaminopyridine or triethylamine were used (entries 4–6). In the case of 2,6-lutidine, some isocyanate 2 was generated after 30 min at rt, but the final product 4a was formed in only 16% yield (entry 7). Neither acetic anhydride nor trifluoroacetic anhydride were able to promote the formation of isocyanate 2 regardless of the temperature applied.

In so far reported procedures for the one-pot syntheses of ureas from carbamates, the use of an excess of amine substrate, usually up to 3 equivalents (or more for less nucleophilic amines) is recommended to obtain the higher efficiency of the process.^48,50,51^ However, in the case of t^6^A/ms^2^t^6^A synthesis, the amine nucleoside substrate, especially non-native 2-methylthioadenosine (ms^2^A) is a very costly reagent. Therefore, in the second step of optimizations we examined an excess of *N*-Boc protected l-threonine derivative 1 to nucleoside 3a in a range 1.5–2.5 (entries 8–10), yet the concentrations of Tf_2_O and 2-Cl-Py against 1 were kept as determined previously (entry 2). We were glad to see that 1.5 molar excess of 1 to 3a led to a significantly better yield of 4a (71%, entry 8), while very high conversion of 3a to 4a was observed when 2.5 equivalents of 1 was applied (92%, entry 10). Unfortunately, further increase in the excess of 1 (3 equiv. or more) did not lead to a higher isolated yield of product 4a. Finally, the use of toluene instead of dichloromethane for the formation of 2 allowed us to carry out the whole process in the same solvent (92% yield, entry 11) which facilitate the preparative procedure for the one-pot synthesis of t^6^A derivative 4a.

**Scheme 2:**
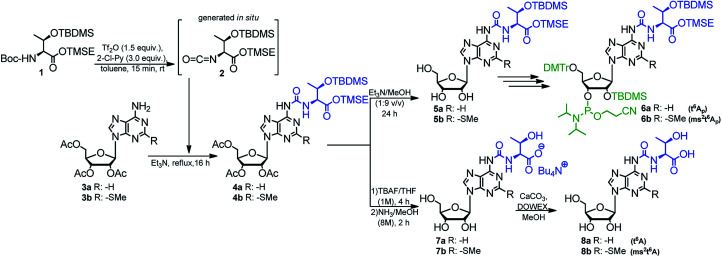
Preparation of t^6^A and ms^2^t^6^A 3′-*O*-phosphoramidities and samples of modified nucleosides t^6^A and ms^2^t^6^A.

The optimized method described above was used in synthesis of the phosphoramidite derivatives of t^6^A and ms^2^t^6^A (6a, and 6b, respectively; Scheme 2). Starting from 2.5 mmol of appropriately protected Boc-l-threonine 1^39^ and 1 mmol of adenosine derivative 3a^57^ or 3b,^39^ the modified nucleosides 4a and 4b were obtained in 92% and 86% yield, respectively. Next, the acetyl groups in 4a/4b were removed under conditions safe for the installed *N*^6^-threonylcarbamoyl chain (Et_3_N/MeOH, rt, 24 h) and the resultant 5a/5b were appropriately protected and phosphitylated according to the previously reported procedures^39^ to give t^6^A/ms^2^t^6^A-phosphoramidites (6a/6b) in 48% and 42% overall yield, respectively (see ESI† for details).

**Fig. 2:**
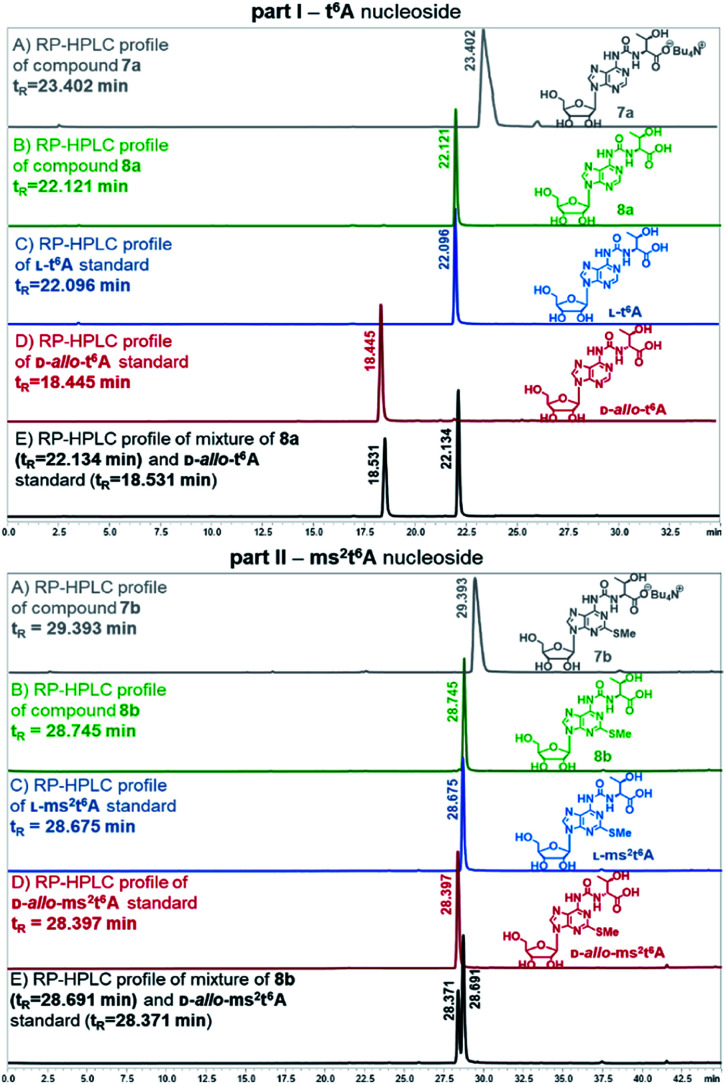
HPLC profiles recorded for l-t^6^A and l-ms^2^t^6^A nucleosides and the corresponding d-*allo* nucleoside standards (d-*allo*-t^6^A, d-*allo*-ms^2^t^6^A).

Also, the nucleosides 4a/4b were deprotected to yield 8a/8b (Scheme 2), to be used as standards in analysis of enzymic hydrolysates of t^6^A- or ms^2^t^6^A-containing oligomers. The silyl protecting groups (TBDMS, TMSE) were removed with excess 1 M tetrabutylammonium fluoride (TBAF) in THF (4 h, rt), and the acetyl groups were cleaved off with NH_3_/MeOH (2 h, rt) (see experimental details in ESI†). The reactions were virtually quantitative and the HPLC profiles recorded for the reaction mixtures (Fig. 2) contained single, slightly tailing peaks (profiles in panels (A), part I for t^6^A and part II for ms^2^t^6^A). The tailing was not observed, when the highly lipophilic tetrabutylammonium cations were replaced with H^+^ ions using DOWEX, H^+^/CaCO_3_ treatment.^58^ The resultant acidic forms 8a/8b had the same HPLC mobility as genuine l-t^6^A/l-ms^2^t^6^A standards^21,22,38,39^ (compare profiles in panels (B) and (C)). The profiles recorded for 8a co-injected with d-*allo*-t^6^A^21,38^ and for 8b co-injected with d-*allo*-ms^2^t^6^A^22,39^ (panels (E)) indicate that the new procedure for ureido linkage formation is safe in terms of the stereochemistry at the Cα of the amino acid component. The profiles for the d-*allo* nucleoside standards are shown in panels (D).

**Scheme 3:**
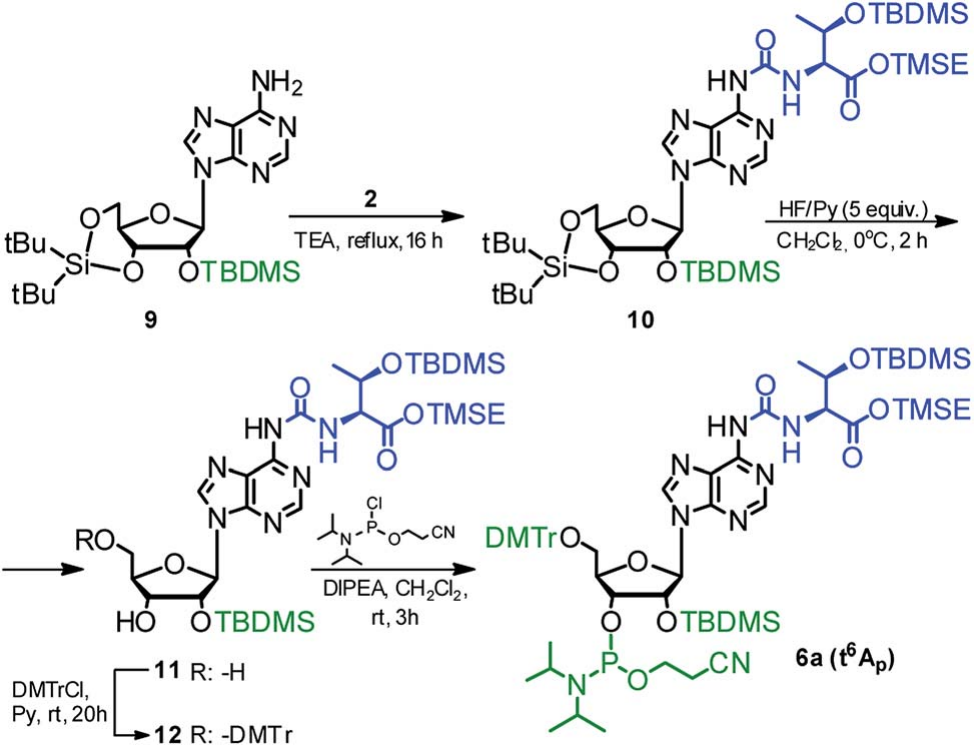
Synthesis of t^6^A phosphoramidite (6a) using 2′-*O*-(*tert*-butyldimethylsilyl)-3′,5′-*O*-(di-*tert*-butylsilylene)adenosine as the nucleoside substrate.

The phosphoramidite derivative of t^6^A (6a) was also synthesized using 2′-*O*-(*tert*-butyldimethylsilyl)-3′,5′-*O*-(di-*tert*-butylsilylene) protected adenosine 9^54,59^ as the nucleoside substrate (Scheme 3). The one-pot conversion 9 → 10 proceeded in 94% yield. Subsequent selective removal of the cyclic silyl protecting group (HF in pyridine, 0 °C) furnished compound 11 (96% yield), further converted into the 5′-*O*-DMTr derivative 12 (90% yield). The reaction of 12 with 2-cyanoethyl *N*,*N*-diisopropylchloro-phosphoramidite gave finally the target t^6^A-phosphoramidite in 91% yield (combined yield 74% for 9 → 6a).

## Conclusions

The presented here modification of the isocyanate method of formation of the ureido linkage between adenosine and threonine greatly facilitates synthesis of fully protected l-threonylcarbamoyl modified adenosines 4a,b rendering subsequent preparation of the t^6^A/ms^2^t^6^A phosphoramidite monomers 6a,b much more efficient. The developed one-pot procedure for 4a,b synthesis, consisting in the epimerization-free formation of l-threonine isocyanate directly from the *N*-Boc-Thr upon activation with Tf_2_O in the presence of 2-Cl-Py, followed by its straight reaction with the *N*^6^*exo*-amine function of the sugar protected nucleoside, eliminates the use of toxic phosgene and provides a shorter protocol for the preparation of the protected t^6^A/ms^2^t^6^A derivatives compared to the previously reported isocyanate and carbamate routes. In addition, the protected nucleosides 4a,b were efficiently deprotected yielding free nucleosides 8a,b to be used as the standards, *e.g.* in HPLC analysis of enzymatically digested oligomers bearing t^6^A/ms^2^t^6^A units. Moreover, the *in situ* formed threonine isocyanate reacted efficiently with 2′-*O*-(*tert*-butyldimethyl-silyl)-3′,5′-*O*-(di-*tert*-butylsilylene)adenosine and the resultant conjugate was conveniently transformed into the t^6^A-phosphoramidite in a very good overall yield 74%. Developed procedures for the synthesis of t^6^A/ms^2^t^6^A 3′-*O*-phosphoramidities will significantly facilitate the availability of monomeric units for the chemical synthesis of various model tRNA fragments suitable for the structure–activity-relationship and biological studies of the t^6^A family nucleosides.

## Experimental

### General remarks

Commercial reagents and analytical grade solvents were used without additional purification unless otherwise stated. Analytical thin layer chromatography (TLC) was done on silica gel coated plates (60 F254, Supelco) with UV light (254 nm) or the ninhydrin test (for amino acids) detection. The products were purified by chromatography on a silica gel 60 (mesh 230–400, Fluka) column eluted with the indicated solvent mixtures. NMR spectra were recorded using a 700 MHz (for ^1^H) instrument, 176 MHz for ^13^C and 283 MHz for ^31^P. Chemical shifts (*δ*) are reported in ppm relative to residual solvent signals CDCl_3_: 7.26 ppm for ^1^H NMR, 77.16 ppm for ^13^C NMR; DMSO-*d*_6_: 2.50 ppm for ^1^H NMR, 39.52 ppm for ^13^C NMR. The signal multiplicities are described as s (singlet), d (doublet), dd (doublet of doublets), ddd (doublets of doublets of doublets), dq (dublet of quartets), t (triplet), td (triplet of doublets), q (quartet), qd (quartet of doublets), m (multiplet), and br s (broad singlet). High-resolution mass spectra were recorded on Synapt G2Si mass spectrometer (Waters) equipped with an ESI source and quadrupole-time-of-flight mass analyzer. HPLC analysis of nucleosides was performed on a Shimadzu Prominence HPLC system equipped with an SPD-M20A spectral photodiode array detector using a Kinetex® column (RP, C18, 5 μm, 4.6 × 250 mm, 100 Å, Phenomenex). Analyses were run at 30 °C and the elution profiles were UV monitored at *λ* = 254 nm.

### General procedure for the one-pot synthesis of 4a, 4b and 10 from Boc-l-threonine 1

To a stirred solution of Boc-l-threonine 1 (1.08 g, 2.5 mmol) in dry toluene (30 mL) 2-chloropyridine (2-Cl-Py, 0.7 mL, 7.5 mmol) was added, followed by trifluoromethanesulfonic anhydride (Tf_2_O, 0.64 mL, 3.75 mmol) and after stirring for 15 min at room temperature triethylamine (Et_3_N, 1.04 mL, 7.5 mmol) and sugar-protected adenosine (3a, 3b or 9, 1.0 mmol) were added. The reaction mixture was stirred under reflux for 16 h. Then the solvent was evaporated under reduced pressure and 4a,b or 10 were isolated by silica gel column chromatography.

#### One-pot synthesis of 4a from Boc-l-threonine 1 and adenosine derivatives 3a

Starting with 2′,3′,5′-tri-*O*-acetyladenosine 3a (0.39 g, 1.0 mmol) 4a was obtained as a white solid in 92% yield (0.69 g, 0.92 mmol) after purification by silica gel column chromatography (0–2% MeOH in CHCl_3_). TLC: *R*_f_ = 0.53 (CHCl_3_/MeOH, 95 : 5 v/v).

^1^H NMR (700 MHz, DMSO-*d*_6_) *δ*: 9.98 (s, 1H, NH-6), 9.86 (d, 1H, ^3^*J* = 9.2 Hz, NH Thr), 8.65 (s, 1H, H-8), 8.42 (s, 1H, H-2), 6.30 (d, 1H, ^3^*J* = 5.4 Hz, H-1′), 6.07–5.99 (m, 1H, H-2′), 5.64 (dd, 1H, ^3^*J* = 5.9 Hz, ^3^*J* = 4.5 Hz, H-3′), 4.49 (qd, 1H, ^3^*J* = 6.2 Hz, ^3^*J* = 1.8 Hz, CH-β Thr), 4.44–4.37 (m, 3H, CH-α Thr, H-4′, H-5′), 4.30–4.24 (m, 1H, H-5′′), 4.18 (ddd, 1H, ^2^*J* = 11.0 Hz, ^3^*J* = 10.0 Hz, ^3^*J* = 6.8 Hz, O–CH TMSE), 4.12 (ddd, 1H, ^3^*J* = 10.9 Hz, ^3^*J* = 10.0 Hz, ^3^*J* = 6.5 Hz, O–CH TMSE), 2.13 (s, 3H, CH_3_–CO Ac), 2.04 (s, 3H, CH_3_–CO Ac), 2.01 (s, 3H, CH_3_–CO Ac), 1.19 (d, ^3^*J* = 6.3 Hz, 3H, CH_3_ Thr), 1.01–0.92 (m, 2H, Si–CH_2_ TMSE), 0.89 (s, 9H, Si–C(CH_3_)_3_ TBDMS), 0.08 (s, 3H, Si–CH_3_ TBDMS), 0.01 (s, 3H, Si–CH_3_ TBDMS), −0.00 (s, 9H, Si(CH_3_)_3_ TMSE); HRMS (ESI-TOF) *m*/*z*: [M + H]^+^ calcd for C_32_H_53_N_6_O_11_Si_2_ 753.3311; found 753.3307 (see Fig. S8 and S26 in the ESI†).

#### One-pot synthesis of 4b from Boc-l-threonine 1 and adenosine derivatives 3b

Starting with 2′,3′,5′-tri-*O*-acetyl-2-methylthioadenosine 3b (0.44 g, 1.0 mmol) 4b was obtained as white solid in 86% yield (0.68 g, 0.86 mmol) after purification by silica gel column chromatography (0–1% MeOH in CHCl_3_). TLC: *R*_f_ = 0.52 (CHCl_3_/MeOH, 95 : 5 v/v).

^1^H NMR (700 MHz, DMSO-*d*_6_) *δ*: 9.98 (s, 1H, NH-6), 9.24 (d, 1H, ^3^*J* = 8.6 Hz, NH Thr), 8.45 (s, 1H, H-8), 6.24 (d, 1H, ^3^*J* = 4.3 Hz, H-1′), 6.07 (dd, 1H, ^3^*J* = 6.0 Hz, ^3^*J* = 4.2 Hz, H-2′), 5.69 (t, 1H, ^3^*J* = 6.1 Hz, H-3′), 4.46–4.44 (m, 2H, CH-α Thr, CH-β Thr), 4.42 (dd, 1H, ^2^*J* = 12.0 Hz, ^3^*J* = 3.7 Hz, H-5′), 4.40–4.35 (m, 1H, H-4′), 4.24–4.16 (m, 2H, 2H, H-5′′, O–CH TMSE), 4.11 (td, 1H, ^2^*J* = 10.7 Hz, ^3^*J* = 6.2 Hz, O–CH TMSE), 2.58 (s, 3H, S–CH_3_), 2.11 (s, 3H, CH_3_–CO Ac), 2.07 (s, 3H, CH_3_–CO Ac), 1.95 (s, 3H, CH_3_–CO Ac), 1.19 (d, 3H, ^3^*J* = 6.3 Hz, CH_3_ Thr), 1.04–0.93 (m, 2H, Si–CH_2_ TMSE), 0.85 (s, 9H, Si–C(CH_3_)_3_ TBDMS), 0.08 (s, 3H, Si–CH_3_ TBDMS), 0.03 (s, 3H, Si–CH_3_ TBDMS), 0.01 (s, 9H, Si(CH_3_)_3_ TMSE); HRMS (ESI-TOF) *m*/*z*: [M + H]^+^ calcd for C_33_H_55_N_6_O_11_SSi_2_ 799.3188; found 799.3177 (see Fig. S14 and S27 in the ESI†).

### Preparation of nucleoside standards 8a and 8b

Fully-protected adenosine 4a or 4b (0.02 g, 0.03 mmol) was dissolved in 1 M solution of TBAF in THF (0.4 mL, 0.40 mmol) and the reaction mixture was stirred for 4 h at room temperature. After this time NH_3_ in dry MeOH (8 M solution, 0.2 mL) was added for deprotection of all acetyl groups from ribose moiety. The reaction was carried out for 2 h and then NH_3_ was removed under reduced pressure to obtain tetrabutylammonium salts 7a/7b. To exchange Bu_4_N^+^ counterion to H^+^, CaCO_3_ (0.28 g), dry DOWEX 50WX8 H^+^ form (0.84 g) and distilled methanol (0.6 mL) were added and the reaction mixture was stirred for 1 h at room temperature.^58^ After this time the resulting mixture was filtered through Celite plug and washed with MeOH. The filtrate was analysed by HPLC and the presence of fully-deprotected only one isomer of 8a/8b with natural l-threonine residue was confirmed (for 8a*R*_*t*_ = 22.121 min, for 8b*R*_*t*_ = 28.745 min, see Fig. 2 panel (B)).

RP-HPLC conditions for analysis of t^6^A derivatives: C18 column with linear gradient of buffer A (0.1% AcOH in H_2_O) and buffer B (ACN) with a flow of 1 mL min^−1^ as follows: 0–15 min from 2% to 8% B, 15–30 min from 8% to 25% B, 30–35 min 2% B. RP-HPLC conditions for analysis of ms^2^t^6^A derivatives: C18 column with linear gradient of buffer A (0.1% AcOH in H_2_O) and buffer B (ACN) with a flow of 1 mL min^−1^ as follows: 0–30 min from 2% B to 15% B, 30–40 min from 15% B to 30% B, 40–45 min 2% B.

### Synthesis of t^6^A 3′-*O*-phoshoramidite 6a from 9

#### One-pot synthesis of 10 from Boc-l-threonine 1 and adenosine derivatives 9

Compound 10 was prepared using 2′-*O*-(*tert*-butyldimethylsilyl)-3′,5′-*O*-(di-*tert*-butylsilylene)-adenosine 9 (0.52 g, 1.0 mmol) according to general one-pot procedure. The crude product 10 was purified by silica gel column chromatography (0–1% MeOH in CHCl_3_) to obtain pure 10 as white solid with 94% yield (0.83 g, 0.94 mmol). TLC: *R*_f_ = 0.68 (CHCl_3_/MeOH, 95 : 5 v/v).

^1^H NMR (700 MHz, CDCl_3_) *δ*: 10.08 (d, 1H, ^3^*J* = 9.1 Hz, NH Thr), 8.49 (s, 1H, H-2), 8.43 (br s, 1H, NH-6), 8.14 (s, 1H, H-8), 5.97 (br s, 1H, H-1′), 4.61–4.56 (m, 3H, H-2′, CH-β Thr, CH-α Thr), 4.52–4.47 (m, 2H, H-3′, H-5′), 4.29–4.21 (m, 2H, H4′, O–CH TMSE), 4.21–4.15 (m, 1H, O–CH_2_ TMSE), 4.06 (dd, ^2^*J* = 10.5 Hz, ^3^*J* = 9.3 Hz, 1H, H5′′), 1.26 (d, 3H, ^3^*J* = 6.3 Hz, CH_3_ Thr), 1.09 (s, 9H, Si–C(CH_3_)_3_*t*Bu_2_Si), 1.05 (s, 9H, Si–C(CH_3_)_3_*t*Bu_2_Si), 1.02–0.99 (m, 2H, Si–CH_2_ TMSE), 0.95 (s, 9H, Si–C(CH_3_)_3_ TBDMS), 0.94 (s, 9H, Si–C(CH_3_)_3_ TBDMS), 0.17 (s, 3H, Si–CH_3_ TBDMS), 0.15 (s, 3H, Si–CH_3_ TBDMS), 0.10 (s, 3H, Si–CH_3_ TBDMS), 0.05 (s, 3H, Si–CH_3_ TBDMS), 0.02 (s, 9H, Si(CH_3_)_3_ TMSE); ^13^C NMR (176 MHz, CDCl_3_) *δ*: 171.16 (C=O Thr), 154.44 (NH–C̲O–NH), 151.48 (C-2), 150.61 (C-6), 149.82 (C-4), 141.31 (C-8), 121.25 (C-5), 92.45 (C-1′), 76.01 (C-3′), 75.74 (C-2′), 74.93 (C-4′), 68.97 (Cα Thr), 67.91 (C-5′), 63.81 (O–CH_2_ TMSE), 59.75 (Cβ Thr), 27.64 (Si–C(C̲H_3_)_3_*t*Bu_2_Si), 27.19 (Si–C(C̲H_3_)_3_*t*Bu_2_Si), 26.05 (Si–C(C̲H_3_)_3_ TBDMS), 25.75 (Si–C(C̲H_3_)_3_ TBDMS), 22.88 (Si–C̲(CH_3_)_3_*t*Bu_2_Si), 21.32 (CH_3_ Thr), 20.51 (Si–C̲(CH_3_)_3_*t*Bu_2_Si), 18.47 (Si–C̲(CH_3_)_3_ TBDMS), 18.03 (Si–C̲(CH_3_)_3_ TBDMS), 17.51 (Si–CH_2_ TMSE), −1.41(Si(CH_3_)_3_ TMSE), −4.07 (Si–CH_3_ TBDMS), −4.15 (Si–CH_3_ TBDMS), −4.84 (Si–CH_3_ TBDMS), −5.15 (Si–CH_3_ TBDMS); HRMS (ESI-TOF) *m*/*z*: [M + H]^+^ calcd for C_40_H_77_N_6_O_8_Si_4_ 881.4880; found 881.4868 (see Fig. S20, S21 and S28 in the ESI†).

#### Preparation of 11 by removal of 3′,5′-*O*-di-*tert*-butyl silylether protection from 10

Fully-protected nucleoside 10 (0.72 g, 0.84 mmol) was dissolved in anhydrous CH_2_Cl_2_ (7.2 mL) and cooled to 0 °C. Then a mixture of 70% HF in pyridine (0.1 mL, 4.2 mmol) and anhydrous pyridine (0.66 mL) was cooled to 0 °C and added to the reaction mixture. After 2 h stirring at 0 °C the mixture was diluted with CH_2_Cl_2_ (15 mL) and extracted with saturated NaHCO_3_ (3 × 15 mL). The organic layer was dried over anhydrous MgSO_4_, filtered and evaporated under reduced pressure. The oily residue was co-evaporated with toluene (2 × 15 mL) and silica gel column chromatography (0–1% MeOH in CH_2_Cl_2_) furnished 11 in 96% yield (0.60 g, 0.81 mmol). TLC: *R*_f_ = 0.48 (CHCl_3_/MeOH, 95 : 5 v/v).

^1^H NMR (700 MHz, DMSO-*d*_6_) *δ*: 9.92 (s, 1H, NH-6), 9.88 (d, 1H, ^3^*J* = 9.0 Hz, NH Thr), 8.71 (s, 1H, H-8), 8.39 (s, 1H, H-2), 6.03 (d, 1H, ^3^*J* = 5.6 Hz, H-1′), 5.18 (dd, 1H, ^3^*J* = 6.3 Hz, ^3^*J* = 5.1 Hz, 5′OH), 5.13 (d, 1H, ^3^*J* = 5.3 Hz, 3′OH), 4.71 (dd, 1H, ^3^*J* = 5.7 Hz, ^3^*J* = 4.8 Hz, H2′), 4.48 (qd, 1H, ^3^*J* = 6.2 Hz, ^3^*J* = 1.9 Hz, CH-β Thr), 4.41 (dd, 1H, ^3^*J* = 9.0 Hz, ^3^*J* = 2.0 Hz, CH-α Thr), 4.20–4.15 (m, 2H, H3′, O–CH TMSE), 4.12 (ddd, 1H, ^2^*J* = 11.0 Hz, ^3^*J* = 9.9 Hz, ^3^*J* = 6.6 Hz, O–CH TMSE), 4.02 (q, 1H, ^3^*J* = 3.7 Hz, H4′), 3.74 (ddd, 1H, ^2^*J* = 12.1 Hz, ^3^*J* = 5.1 Hz, ^3^*J* = 4.0 Hz, H5′), 3.74 (ddd, 1H, ^2^*J* = 12.1 Hz, ^3^*J* = 6.3 Hz, ^3^*J* = 3.6 Hz, H5′′), 1.19 (d, ^3^*J* = 6.3 Hz, 3H, CH_3_ Thr), 0.99–0.91 (m, 2H, Si–CH_2_ TMSE), 0.88 (s, 9H, Si–C(CH_3_)_3_ TBDMS), 0.72 (s, 9H, Si–C(CH_3_)_3_ TBDMS), 0.07 (s, 3H, Si–CH_3_ TBDMS), 0.00 (s, 3H, Si–CH_3_ TBDMS), −0.02 (s, 9H, Si–(CH_3_)_3_ TMSE), −0.07 (s, 3H, Si–CH_3_ TBDMS), −0.18 (s, 3H, Si–CH_3_ TBDMS); ^13^C NMR (176 MHz, DMSO-*d*_6_) *δ*: 170.62 (C=O Thr), 153.68 (NH–C̲O–NH), 150.34 (C-6), 150.13 (C-4), 150.10 (C-2), 142.27 (C-8), 120.55 (C-5), 87.90 (C-1′), 85.98 (C-4′), 75.66 (C-2′), 70.12 (C-3′), 68.36 (Cβ Thr), 62.85 (O–CH_2_ TMSE), 61.06 (C-5′), 58.90 (Cα Thr), 25.47 (Si–C(C̲H_3_)_3_ TBDMS), 25.34 (Si–C(C̲H_3_)_3_ TBDMS), 20.86 (CH_3_ Thr), 17.73 (Si–C̲(CH_3_)_3_ TBDMS), 17.47 (Si–C̲(CH_3_)_3_ TBDMS), 16.69 (Si–CH_2_ TMSE), −1.62 (Si(C̲H_3_)_3_ TMSE), −4.35 (Si–CH_3_ TBDMS), −4.94 (Si–CH_3_ TBDMS), −5.42 (Si–CH_3_ TBDMS), −5.59 (Si–CH_3_ TBDMS); HRMS (ESI-TOF) *m*/*z*: [M + H]^+^ calcd for C_32_H_61_N_6_O_8_Si_3_ 741.3859; found 741.3860 (see Fig. S22, S23 and S29 in the ESI†).

#### Preparation of 12 by 5′-*O*-dimethoxytritylation of 11

To a stirred solution of nucleoside 11 (0.55 g, 0.74 mmol) in dry pyridine (6.0 mL) DMTrCl (0.36 g, 0.96 mmol) was added. The reaction was stirred for 20 h at room temperature. The reaction mixture was cooled to 0 °C in an ice bath and quenched with H_2_O (10 mL) and stirred at 0 °C for 15 min. The mixture was extracted with CH_2_Cl_2_ (3 × 15 mL) and the organic layer was dried over anhydrous MgSO_4_, filtered and the solvent was evaporated. The oily residue was co-evaporated with toluene (2 × 10 mL). Silica gel column chromatography (0–1% MeOH in CH_2_Cl_2_) furnished 12 as a white solid in 90% yield (0.69 g, 0.67 mmol). TLC: *R*_f_ = 0.38 (CHCl_3_/MeOH, 98 : 2 v/v).

^1^H NMR (700 MHz, DMSO-*d*_6_) *δ*: 9.92–9.91 (m, 2H, NH-6, NH Thr), 8.59 (s, 1H, H-8), 8.28 (s, 1H, H-2), 7.42–7.38 (m, 2H, H_Ar_ DMTr), 7.29–7.22 (m, 6H, H_Ar_ DMTr), 7.22–7.16 (m, 1H, H_Ar_ DMTr), 6.84–6.79 (m, 4H, H_Ar_ DMTr), 6.02 (d, 1H, ^3^*J* = 5.3 Hz, H-1′), 5.17 (d, 1H, ^3^*J* = 5.7 Hz, 3′OH), 5.01 (t, 1H, ^3^*J* = 5.2 Hz, H-2′), 4.48 (qd, 1H, ^3^*J* = 6.3 Hz, ^3^*J* = 2.0 Hz, CH-β Thr), 4.41 (dd, 1H, ^3^*J* = 9.0 Hz, ^3^*J* = 1.9 Hz, CH-α Thr), 4.29–4.23 (m, 1H, H-3′), 4.18–4.15 (m, 1H, O–CH TMSE), 4.15–4.08 (m, 2H, H-4′, O–CH TMSE), 3.71 (s, 6H, 2× O–CH_3_ DMTr), 3.32 (dd, 1H, ^2^*J* = 10.6 Hz, ^3^*J* = 3.9 Hz, H-5′), 3.25 (dd, 1H, ^2^*J* = 10.5 Hz, ^3^*J* = 5.1 Hz, H-5′′), 1.19 (d, 3H, ^3^*J* = 6.2 Hz, CH_3_ Thr), 1.00–0.89 (m, 2H, Si–CH_2_ TMSE), 0.85 (s, 9H, Si–C(CH_3_)_3_ TBDMS), 0.73 (s, 9H, Si–C(CH_3_)_3_ TBDMS), 0.06 (s, 3H, Si–CH_3_ TBDMS), 0.00 (s, 3H, Si–CH_3_ TBDMS), −0.02 (s, 9H, Si(CH_3_)_3_ TMSE), −0.05 (s, 3H, Si–CH_3_ TBDMS), −0.16 (s, 3H, Si–CH_3_ TBDMS); ^13^C NMR (176 MHz, DMSO-*d*_6_) *δ*: 171.17 (C=O), 158.51 (C_Ar_ DMTr), 154.23 (NH–CO–NH), 150.86 (C-6), 150.59 (C-4), 150.47 (C-2), 145.35 (C_Ar_ DMTr), 143.44 (C-8), 135.95 (C_Ar_ DMTr), 130.18 (C_Ar_ DMTr), 128.17 (C_Ar_ DMTr), 128.15 (C_Ar_ DMTr), 127.05 (C_Ar_ DMTr), 121.26 (C-5), 113.52 (C_Ar_ DMTr), 88.89 (C-1′), 86.02 ( <svg xmlns="http://www.w3.org/2000/svg" version="1.0" width="10.400000pt" height="16.000000pt" viewBox="0 0 10.400000 16.000000" preserveAspectRatio="xMidYMid meet"><metadata> Created by potrace 1.16, written by Peter Selinger 2001-2019 </metadata><g transform="translate(1.000000,15.000000) scale(0.011667,-0.011667)" fill="currentColor" stroke="none"><path d="M80 1160 l0 -40 40 0 40 0 0 -40 0 -40 40 0 40 0 0 -40 0 -40 40 0 40 0 0 -40 0 -40 40 0 40 0 0 -40 0 -40 40 0 40 0 0 -40 0 -40 40 0 40 0 0 -40 0 -40 40 0 40 0 0 80 0 80 -40 0 -40 0 0 40 0 40 -40 0 -40 0 0 40 0 40 -40 0 -40 0 0 40 0 40 -40 0 -40 0 0 40 0 40 -40 0 -40 0 0 40 0 40 -80 0 -80 0 0 -40z M560 520 l0 -40 -40 0 -40 0 0 -40 0 -40 -40 0 -40 0 0 -40 0 -40 -40 0 -40 0 0 -40 0 -40 -40 0 -40 0 0 -40 0 -40 -40 0 -40 0 0 -40 0 -40 -40 0 -40 0 0 -40 0 -40 80 0 80 0 0 40 0 40 40 0 40 0 0 40 0 40 40 0 40 0 0 40 0 40 40 0 40 0 0 40 0 40 40 0 40 0 0 40 0 40 40 0 40 0 0 80 0 80 -40 0 -40 0 0 -40z"/></g></svg> C <svg xmlns="http://www.w3.org/2000/svg" version="1.0" width="10.400000pt" height="16.000000pt" viewBox="0 0 10.400000 16.000000" preserveAspectRatio="xMidYMid meet"><metadata> Created by potrace 1.16, written by Peter Selinger 2001-2019 </metadata><g transform="translate(1.000000,15.000000) scale(0.011667,-0.011667)" fill="currentColor" stroke="none"><path d="M480 1160 l0 -40 -40 0 -40 0 0 -40 0 -40 -40 0 -40 0 0 -40 0 -40 -40 0 -40 0 0 -40 0 -40 -40 0 -40 0 0 -40 0 -40 -40 0 -40 0 0 -80 0 -80 40 0 40 0 0 40 0 40 40 0 40 0 0 40 0 40 40 0 40 0 0 40 0 40 40 0 40 0 0 40 0 40 40 0 40 0 0 40 0 40 40 0 40 0 0 40 0 40 40 0 40 0 0 40 0 40 -80 0 -80 0 0 -40z M80 480 l0 -80 40 0 40 0 0 -40 0 -40 40 0 40 0 0 -40 0 -40 40 0 40 0 0 -40 0 -40 40 0 40 0 0 -40 0 -40 40 0 40 0 0 -40 0 -40 80 0 80 0 0 40 0 40 -40 0 -40 0 0 40 0 40 -40 0 -40 0 0 40 0 40 -40 0 -40 0 0 40 0 40 -40 0 -40 0 0 40 0 40 -40 0 -40 0 0 40 0 40 -40 0 -40 0 0 40 0 40 -40 0 -40 0 0 -80z"/></g></svg> DMTr), 84.38 (C-4′), 74.84 (C-2′), 70.69 (C-3′), 68.86 (C-β), 63.84 (C-5′), 63.36 (O–CH_2_ TMSE), 59.40 (C-α), 55.44 (O–CH_3_ DMTr), 25.99 (C–Si–(CH_3_)_3_ TBDMS), 25.79 (C–Si–(CH_3_)_3_ TBDMS), 21.35 (CH_3_), 18.25 (C–Si–(CH_3_)_3_ TBDMS), 17.93 (C–Si–(CH_3_)_3_ TBDMS), 17.16 (Si–CH_2_ TMSE), −1.11 (Si–(CH_3_)_3_ TMSE), −3.90 (Si–CH_3_ TBDMS), −4.36 (Si–CH_3_ TBDMS), −4.86 (Si–CH_3_ TBDMS), −5.10 (Si–CH_3_ TBDMS); HRMS (ESI-TOF) *m*/*z*: [M + H]^+^ calcd for C_53_H_79_N_6_O_10_Si_3_ 1043.5165; found 1043.5170 (see Fig. S24, S25 and S30 in the ESI†).

#### Preparation of 6a by 3′-*O*-phosphitylation of 12

3′-*O*-Phoshoramidite 6a was prepared according to the literature procedure,^41^ using compound 12 (0.60 g, 0.56 mmol), 2-cyanoethyl-*N*,*N*-diisopropylchlorophosphoramidite (0.24 mL, 1.12 mmol), DIPEA (0.4 mL, 2.24 mmol) and freshly distilled CH_2_Cl_2_ (3.2 mL). The crude product was purified by the flash chromatography (silica gel, petroleum ether/acetone, 2 : 1 v/v) to obtain pure product 6a in 92% yield (0.64 g, 0.52 mmol). TLC: *R*_f_ = 0.52 (CHCl_3_/acetone, 95 : 5 v/v).

^31^P NMR: (283 MHz, C_6_H_6_) *δ*: 149.89, 148.04 (see Fig. S12 in the ESI†).

## Conflicts of interest

There are no conflicts to declare.

## Supplementary Material

RA-011-D0RA09803E-s001

## References

[cit1] Boccaletto P., Machnicka M. A., Purta E., Piatkowski P., Baginski B., Wirecki K., de Crecy-Lagard V., Ross R., Limbach P. A., Kotter A., Helm M., Bujnicki J. M. (2018). Nucleic Acids Res..

[cit2] Cantara W. A., Crain P. F., Rozenski J., McCloskey J. A., Harris K. A., Zhang X., Vendeix F. A., Fabris D., Agris P. F. (2011). Nucleic Acids Res..

[cit3] McCown P. J., Ruszkowska A., Kunkler C. N., Berger K., Hulewicz J. P., Wang M. C., Springer N. A., Brown J. A. (2020). Wiley Interdiscip. Rev.: RNA.

[cit4] Väre V., Eruysal E., Narendran A., Sarachan K., Agris P. (2017). Biomolecules.

[cit5] Helm M., Alfonzo J. D. (2014). Chem. Biol..

[cit6] Björk G. R., Hagervall T. G. (2014). EcoSal Plus.

[cit7] Machnicka M. A., Olchowik A., Grosjean H., Bujnicki J. M. (2014). RNA Biol..

[cit8] Pollo-Oliveira L., de Crécy-Lagard V. (2019). Biochemistry.

[cit9] El Yacoubi B., Bailly M., de Crécy-Lagard V. (2012). Annu. Rev. Genet..

[cit10] Tuorto F., Lyko F. (2016). Open Biol..

[cit11] Duechler M., Leszczyńska G., Sochacka E., Nawrot B. (2016). Cell. Mol. Life Sci..

[cit12] Endres L., Dedon P. C., Begley T. J. (2015). RNA Biol..

[cit13] Torres A. G. T., Batlle E., Ribas de Pouplana L. (2014). Trends Mol. Med..

[cit14] Machnicka M. A., Olchowik A., Grosjean H., Bujnicki J. M. (2014). RNA Biol..

[cit15] Pichard-Kostuch A. , Daugeron M.-C., Forterre P. and Basta T., in RNA metabolism and gene expression in Archaea, ed. B. Clouet d'Orval, Springer International Publishing AG, 2017, ch. 8, pp. 177–200

[cit16] Thiaville P. C., El Yacoubi B., Köhrer C., Thiaville J. J., Deutsch C., Iwata-Reuyl D., Bacusmo J. M., Armengaud J., Bessho Y., Wetzel C., Cao X., Limbach P. A., RajBhandary U. L., de Crecy-Lagard V. (2015). Mol. Microbiol..

[cit17] Schweizer M. P., Chheda G. B., Baczynskyj L., Hall R. H. (1969). Biochemistry.

[cit18] Yamaizumi Z., Nishimura S., Limburg K., Raba M., Gross H. J., Crain P. F., McCloskey J. M. (1979). J. Am. Chem. Soc..

[cit19] Kimura-Harada F., von Minden D. L., McCloskey J. A., Nishimura S. (1972). Biochemistry.

[cit20] Miyauchi K., Kimura S., Suzuki T. (2013). Nat. Chem. Biol..

[cit21] Matuszewski M., Wojciechowski J., Miyauchi K., Gdaniec Z., Wolf W. M., Suzuki T., Sochacka E. (2017). Nucleic Acids Res..

[cit22] Kang B., Miyauchi K., Matuszewski M., D'Almeida G. S., Rubio M. A. T., Alfonzo J. D., Inoue K., Sakaguchi Y., Suzuki T., Sochacka E., Suzuki T. (2017). Nucleic Acids Res..

[cit23] Nagao A., Ohara M., Miyauchi K., Yokobori S. I., Yamagishi A., Watanabe K., Suzuki T. (2017). Nat. Struct. Mol. Biol..

[cit24] Chheda G. B., Hong C. I. (1971). J. Med. Chem..

[cit25] Hong C. I. and Chheda G. B., in Nucleic Acid Chemistry, ed. L. B. Towsend and R. S. Tipson, John Wiley & Sons, New York, 1972, pp. 661–664

[cit26] Hong C. I., Chheda G. B., Dutta S. P., Grady-Curtis A. O., Tritsch G. L. (1973). J. Med. Chem..

[cit27] Adamiak R. W., Wiewiórowski M. (1974). Bull. Acad. Pol. Sci., Ser. Sci. Chim..

[cit28] Martin D., Schlimme E. (1994). Z. Naturforsch., C: J. Biosci..

[cit29] Sundaram M., Crain P. F., Davis D. R. (2000). J. Org. Chem..

[cit30] Lyon P. A., Reese C. B. (1978). J. Chem. Soc., Perkin Trans. 1.

[cit31] Adamiak R. W., Stawinski J. (1977). Tetrahedron Lett..

[cit32] Adamiak R. W., Biala E., Grzeskowiak K., Kierzek R., Kraszewski A., Markiewicz W. T., Okupniak J., Stawinski J., Wiewiorowski M. (1978). Nucleic Acids Res..

[cit33] Sochacka E. (1998). Nucleosides Nucleotides.

[cit34] Leszczynska G., Pieta J., Sproat B., Małkiewicz A. (2011). Tetrahedron Lett..

[cit35] Bajji A. C., Davis D. R. (2002). J. Org. Chem..

[cit36] Baiji C., Sundaram M., Myszka D. G., Davis D. R. (2002). J. Am. Chem. Soc..

[cit37] Davis D. R., Bajji A. C. (2005). Methods Mol. Biol..

[cit38] Matuszewski M., Debiec K., Sochacka E. (2017). Chem. Commun..

[cit39] Debiec K., Matuszewski M., Podskoczyj K., Leszczynska G., Sochacka E. (2019). Chem.–Eur. J..

[cit40] Nainyte M., Muller F., Ganazzoli G., Chan Ch.-Y., Crisp A., Globisch D., Carell T. (2020). Chem.–Eur. J..

[cit41] Himmelsbach F., Schultz B. S., Trichtinger T., Charubala R., Pfleiderer W. (1984). Tetrahedron.

[cit42] Schneider Ch., Becker S., Okamura H., Crisp A., Amatov T., Stadlmeier M., Carell T. (2018). Angew. Chem., Int. Ed..

[cit43] Boudou V., Langridge J., Aerschot A. V., Hendrix C., Millar A., Weiss P., Herdewijn P. (2000). Helv. Chim. Acta.

[cit44] Lamothe M., Perez M., Colovray-Gotteland V., Halazy S. (1996). Synlett.

[cit45] Chong P. Y., Janicki S. Z., Petillo P. A. (1998). J. Org. Chem..

[cit46] Gastaldi S., Weinreb S. M., Stien D. (2000). J. Org. Chem..

[cit47] In J., Hwang S., Kim C., Seo J. H., Kim S. (2013). Eur. J. Org. Chem..

[cit48] Spyropoulos C., Kokotos C. G. (2014). J. Org. Chem..

[cit49] Cho H., Lee J. O., Hwang S., Seo J. H., Kim S. (2016). Asian J. Org. Chem..

[cit50] Kim H.-K., Lee A. (2016). Tetrahedron Lett..

[cit51] Bana P., Lako A., Kiss N. Z., Beni Z., Szigetvari A., Koti J., Turos G. I., Eles J., Greiner I. (2017). Org. Process Res. Dev..

[cit52] Kang S., Kim H.-K. (2018). Tetrahedron.

[cit53] Wang M., Han J., Si X., Hu Y., Zhu J., Sun X. (2018). Tetrahedron Lett..

[cit54] Serebryany V., Beigelman L. (2002). Tetrahedron Lett..

[cit55] Serebryany V., Beigelman L. (2003). Nucleosides, Nucleotides Nucleic Acids.

[cit56] Saito Y., Nyilas A., Agrofoglio L. A. (2001). Carbohydr. Res..

[cit57] Reese C. B., Shek L. H. K., Zhao Z. (1995). J. Chem. Soc., Perkin Trans. 1.

[cit58] Kaburagi Y., Kishi Y. (2007). Org. Lett..

[cit59] Shishodia S., Zhang D., El-Sagheer A. H., Brown T., Claridge T. D. W., Schofield C. J., Hopkinson R. J. (2018). Org. Biomol. Chem..

