# Supplementary material for: Efficient access to 3′-O-phosphoramidite derivatives of tRNA related N6-threonylcarbamoyladenosine (t6A) and 2-methylthio-N6-threonylcarbamoyladenosine (ms2t6A)
Source: RSC Adv. 2021 Jan 7;11(4):1992–9. doi: 10.1039/d0ra09803e (PMC8693639; doi:10.1039/d0ra09803e)

Electronic Supplementary Material (ESI) for RSC Advances.
This journal is © The Royal Society of Chemistry 2020

Electronic Supporting Information

Efficient access to 3'-*O*-phosphoramidite derivatives of tRNA related *N*^{6}-threonylcarbamoyladenosine (t^6A) and 2-methylthio-*N*^{6}-threonylcarbamoyladenosine (ms^2t^6A)

Katarzyna Debiec, Elzbieta Sochacka*

Institute of Organic Chemistry, Faculty of Chemistry, Lodz University of Technology, Zeromskiego 116, 90-924 Lodz, Poland; K.D. e-mail: katarzyna.debiec@dokt.p.lodz.pl; E.S.* e-mail: elzbieta.sochacka@p.lodz.pl

Table of Contents:

I. General remarks 2

II. Experimental details for synthesis 6a, 6b. 3

2.1 Synthesis of t^6A-phosphoramidite 6a from 4a. 3

2.2 Synthesis of ms^2t^6A-phosphoramidite 6b from 4b. 6

III. Spectroscopic data. 9

3.1 NMR and IR spectra of threonine derivatives. 9

3.2 NMR spectra of adenosine derivatives 15

3.3 HR-MS spectra of adenosine derivatives 34

I. General remarks

Commercial reagents and analytical grade solvents were used without additional purification unless otherwise stated. Analytical thin layer chromatography (TLC) was done on silica gel coated plates (60 F254, Supelco) with UV light (254 nm) or the ninhydrin test (for amino acids) detection. The products were purified by column chromatography on a silica gel 60 (mesh 230 – 400, Fluka) eluted with the indicated solvent mixtures. NMR spectra were recorded using a 700 MHz (for ^{1}H) instrument, 176 MHz for ^{13}C and 101 MHz for ^{31}P. Chemical shifts (δ) are reported in ppm relative to residual solvent signals $CDCl_3$: 7.26 ppm for ^{1}H NMR, 77.16 ppm for ^{13}C NMR; DMSO-d_6: 2.50 ppm for ^{1}H NMR, 39.52 ppm for ^{13}C NMR. The signal multiplicities are described as s (singlet), d (doublet), dd (doublet of doublets), ddd (doublet of doublets of doublets), dq (doublet of quartets), t (triplet), td (triplet of doublets), q (quartet), qd (quartet of doublets), m (multiplet), and br s (broad singlet). High-resolution mass spectra were recorded on Synapt G2Si mass spectrometer (Waters) equipped with an ESI source and quadrupole-Time-of-flight mass analyser. IR data were recorded on an FT-IR ALPHA instrument (Bruker) equipped with a Platinum ATR QuickSnap™ module. Analytical HPLC of nucleosides were performed on a Shimadzu Prominence HPLC system equipped with an SPD-M20A spectral photodiode array detector using a Kinetex® column (RP, C18, 5 µm, 4.6 × 250 mm, 100 Å, Phenomenex). Analyses were run at 30 °C and the elution profiles were UV monitored at λ = 254 nm.

II. Experimental details for synthesis 6a, 6b.

2.1 Synthesis of t⁶A-phosphoramidite 6a from 4a.

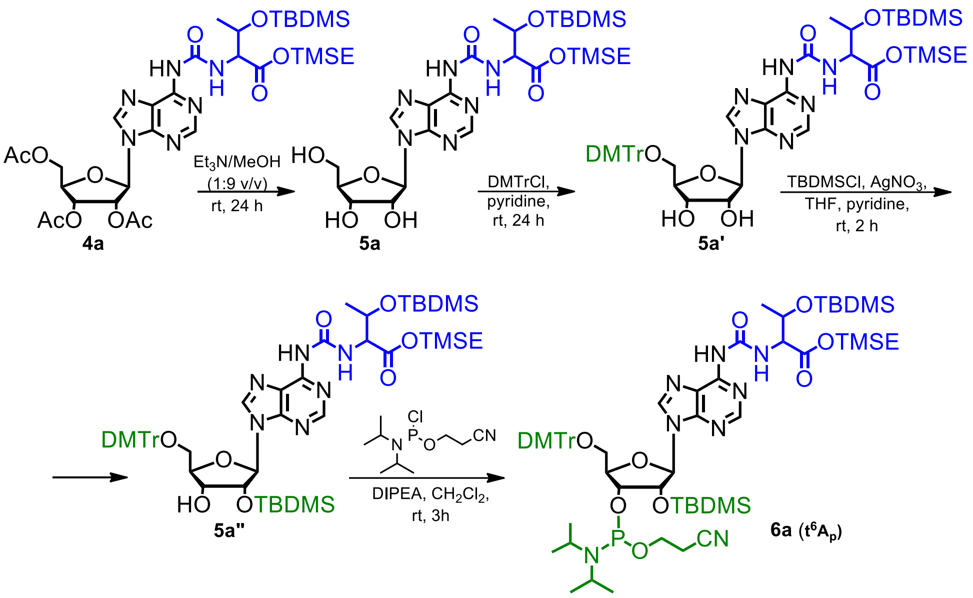

<u>*N*-[[9-(β-D-Ribofuranosyl)-purin-6-yl]carbamoyl]-*O*-*tert*-butyldimethylsilyl-L-threonine trimethylsilylethyl ester (**5a**)</u>

Ref: K. Debiec, M. Matuszewski, K. Podskoczyj, G. Leszczyńska, E. Sochacka, *Chem. Eur. J.*, **2019**, *25*, 13309; F. Himmelsbach, B. S. Schulz, T. Trichtinger, R. Charubala, W. Pfleiderer, *Terahedron*, **1984**, *40*, 59.

To protected adenosine **4a** (0.3 g, 0.40 mmol) the solution of distilled solvents: Et_3N/MeOH (4.5 mL, 1:9 *v/v*) was added. The reaction mixture was stirred for 24 h at room temperature and the solvents were removed under reduced pressure. The oily residue was co-evaporated with toluene (2 x 5 mL). The crude product was purified by column chromatography (silica gel, 0-5 % MeOH in $CHCl_3$) to obtain pure product **5a** in 90 % yield (0.23 g, 0.36 mmol). **TLC:** Rf = 0.21 ($CHCl_3$/MeOH, 95:5 *v/v*);

^{1}H NMR (700 MHz, DMSO-d_6) δ: 9.91-9.89 (m, 2H, NH-6, NH Thr), 8.69 (s, 1H, H-8), 8.41 (s, 1H, H-2), 6.00 (d, 1H, ^3J=5.6 Hz, H-1'), 5.52 (br s, 1H, 2'OH), 5.24 (s, 1H, 3'OH), 5.11 (s, 1H, 5'OH), 4.59 (t, 1H, ^3J=5.2 Hz, H-2'), 4.48 (qd, 1H, ^3J=6.3 Hz, ^3J=1.9 Hz, CH-β Thr), 4.42 (dd, 1H, ^3J=9.0 Hz, ^3J=1.9 Hz, CH-α Thr), 4.21-4.16 (m, 2H, H-3', O-CH TMSE), 4.15-4.07 (m, 1H, O-CH TMSE), 3.98 (q, 1H, ^3J=3.9 Hz, H-4'), 3.70 (dd, 1H, ^2J=12.0 Hz, ^3J=4.0 Hz, H-5'), 3.58 (dd, 1H, ^2J=12.0 Hz, ^3J=4.0 Hz, H-5"), 1.19 (d, 3H, ^3J= 6.3 Hz, CH_3 Thr), 0.99-0.93 (m, 2H, Si-CH_2 TMSE), 0.89 (s, 9H, Si-C$(CH_3)_3$ TBDMS), 0.08 (s, 3H, Si-CH_3 TBDMS), 0.01 (s, 3H, Si-CH_3 TBDMS), -0.00 (s, 9H, Si$(CH_3)_3$ TMSE).

N-[[9-(5'-O-(4,4'-dimethoxytrityl)-β-D-ribofuranosyl)-purin-6-yl]carbamoyl]-O-tert-butyldimethylsilyl-L-threonine trimethylsilylethyl ester (**5a'**)

Ref: M. Sundaram, P. F. Crain, D. R. Davis, *J. Org. Chem.*, **2000**, *65*, 5609;

To a stirred solution of nucleoside **5a** (0.2 g, 0.32 mmol) in dry pyridine (3.0 mL) DMTrCl (0.13 g, 0.38 mmol) was added. The reaction was stirred for 20 h at room temperature. After this time the reaction mixture was cooled to 0°C in an ice bath and quenched with H_2O (4 mL) and stirred at 0°C for 15 min. The mixture was extracted with $CHCl_3$ (2 x 15 mL) and combined organic layers were dried over anhydrous $MgSO_4$, filtered and the solvent was evaporated. The oily residue was co-evaporated with toluene (2 x 8 mL). The crude product was purified by column chromatography (silica gel, 0-1 % MeOH in $CHCl_3$) to obtain **5a'** as a white solid in 90 % yield (0.27 g, 0.29 mmol). **TLC:** R_f = 0.43 ($CHCl_3$/MeOH, 95:5 *v/v*).

^{1}H NMR (700 MHz, DMSO-d_6) δ: 9.93 (d, ^3J=8.9 Hz, 1H, NH Thr), 9.90 (s, 1H, NH-6), 8.58 (s, 1H, H-8), 8.34 (s, 1H, H-2), 7.36-7.33 (m, 2H, H_{Ar} DMTr), 7.24-7.19 (m, 6H, H_{Ar} DMTr), 7.19-7.15 (m, 1H, H_{Ar} DMTr), 6.81-6.78 (m, 4H, H_{Ar} DMTr), 6.03 (d, 1H, ^3J=4.5 Hz, H-1'), 5.61 (br s, 1H, 2'OH), 5.28 (br s, 1H, 3'OH), 4.81 (t, 1H, ^3J= 4.8 Hz, H-2'), 4.50 (qd, 1H, ^3J=6.2 Hz, ^3J=1.9 Hz, CH-β Thr), 4.42 (dd, 1H, ^3J=9.1 Hz, ^3J=1.8 Hz, CH-α Thr), 4.36 (t, 1H, ^3J=5.1 Hz, H-3'), 4.19 (ddd, 1H, ^2J=11.0 Hz, ^3J=10.0 Hz, ^3J=6.8 Hz, O-CH TMSE), 4.15-4.08 (m, 2H, H-4', O-CH TMSE), 3.71 (s, 3H, O-CH_3 DMTr), 3.71 (s, 3H, O-CH_3 DMTr), 3.28-3.22 (m, 2H, H-5', H-5"), 1.21 (d, 3H, ^3J=6.3 Hz, CH_3 Thr), 0.99-0.93 (m, 2H, Si-CH_2 TMSE), 0.89 (s, 9H, Si-C(CH_3)$_3$ TBDMS), 0.08 (s, 3H, Si-CH_3 TBDMS), 0.01 (s, 3H, Si-CH_3 TBDMS), -0.01 (s, 9H, Si(CH_3)$_3$ TMSE).

N-[[9-(2′-O-tert-butyldimethylsilyl-5′-O-(4,4′-dimethoxytrityl)-β-D-ribofuranosyl)-purin-6-yl] carbamoyl]-O-tert-butyldimethylsilyl-L-threonine trimethylsilylethyl ester (**5a"**)

Ref: K. Onizuka, M.E. Hazemi, J.M. Thomas , L.R. Monteleone, K. Yamada, S. Imoto, P.A. Beal, F. Nagatsugi, *Bioorg Med Chem.*, **2017**, *25*, 2191; V. Boudou, J. Langridge, A. V. Aerschot, C. Hendrix, A. Millar, P. Weiss, P. Herdewijn, Helv. Chim. Acta, **2000**, *83*, 152;

5′-DMTr nucleoside **5a'** (0.2 mg, 0.22 mmol) was dissolved in freshly distilled THF (1.0 mL) then anhydrous pyridine (0.09 mL, 1.1 mmol) and $AgNO_3$ (56 mg, 0.33 mmol) was added. The reaction mixture was stirred at room temperature for 30 min in darkness. Then TBDMS-Cl (0.06 g, 0.4 mmol) was added and stirring was continued at room temperature for 2 h. After this time, the reaction mixture was diluted with CH_2Cl_2, filtered through Celite and washed with CH_2Cl_2. The solution was extracted with saturated $NaHCO_3$ (2 x 10 mL) and brine (10 mL). The organic layer was dried over $MgSO_4$, filtered and concentrated under reduced pressure. The oily residue was

co-evaporated with toluene (2 x 5 mL) and the crude product was purified by the column chromatography (silica gel, 0-10 % acetone in $CHCl_3$). The pure 2'-isomer **5a"** was obtained as a white solid in 57 % yield (0.13 g, 0.13 mmol. The 3'-regioisomer (0.06 g, 0.06 mmol) was dissolved in dry methanol with few drops of TEA, to give equimolar mixture of 2' and 3' TBDMS isomers from which 2'-TBDMS nucleoside was chromatographically isolated to give finally 0.16 g of **5a"** (0.15 mmol; 70 % yield). **TLC:** R_f= 0.42 ($CHCl_3$/MeOH, 98:2 *v/v*).

^{1}H NMR (700 MHz, DMSO-d_6) δ: 9.93 (br s, 1H, NH Thr), 9.92 (s, 1H, NH-6), 8.60 (s, 1H, H-8), 8.28 (s, 1H, H-2), 7.42-7.38 (m, 2H, H_{Ar} DMTr), 7.29-7.26 (m, 6H, H_{Ar} DMTr), 7.26-7.22 (m, 1H, H_{Ar} DMTr), 7.21-7.17 (m, 4H, H_{Ar} DMTr), 6.84-6.80 (m, 4H, H_{Ar} DMTr), 6.03 (d, 1H, ^3J=5.3 Hz, H-1'), 5.17 (d, 1H, ^3J=5.7 Hz, 3'OH), 5.01 (t, 1H, ^3J=5.2 Hz, H-2'), 4.48 (qd, 1H, ^3J=6.3 Hz, ^3J=2.0 Hz, CH-β Thr), 4.42 (dd, 1H, ^3J=9.0 Hz, ^3J=1.9 Hz, CH-α Thr), 4.29-4.24 (m, 1H, H-3'), 4.18 (ddd, 1H, ^3J=11.1 Hz, ^3J=9.8 Hz, ^3J=6.8 Hz, O-CH TMSE), 4.15-4.09 (m, 2H , H-4', O-CH TMSE), 3.71 (s, 6H, 2x O-CH_3 DMTr), 3.34-3.31 (m, 1H, H-5'), 3.26 (dd, 1H, ^2J=10.5 Hz, , ^3J=5.2 Hz, H-5"), 1.19 (d, 3H, ^3J=6.2 Hz, CH_3 Thr), 0.96-0.93 (m, 2H, Si-CH_2 TMSE), 0.85 (s, 9H, Si-C(CH_3)$_3$ TBDMS), 0.73 (s, 9H, Si-C(CH_3)$_3$ TBDMS), 0.06 (s, 3H, Si-CH_3 TBDMS), -0.00 (s, 3H, Si-CH_3 TBDMS), -0.02 (s, 9H, Si(CH_3)$_3$ TMSE), -0.05 (s, 3H, Si-CH_3 TBDMS), -0.16 (s, 3H, Si-CH_3 TBDMS).

N-[[9-(2'-*O*-*tert*-butyldimethylsilyl-3'-(2-cyanoethyl-*N,N*-diisopropylphosphoramidite)-5'-*O*-(4,4'-dimethoxytrityl)-β-D-ribofuranosyl)-purin-6-yl]carbamoyl]-*O*-*tert*-butyldimethylsilyl-*L*-threonine trimethylsilylethyl ester (**6a**)

Ref: V. Boudou, J. Langridge, A. V. Aerschot, C. Hendrix, A. Millar, P. Weiss, P. Herdewijn, Helv. Chim. Acta, **2000**, *83*, 152;

To a stirred solution of 5'-DMTr, 2'-TBDMS-t^{6}A **5a"** (0.15 mg, 0.14 mmol) in freshly distilled CH_2Cl_2 (0.8 mL) anhydrous DIPEA was added (0.1 mL, 0.56 mmol) under argon atmosphere. Then 2-cyanoethyl-*N,N*-diisopropylchlorophosphoramidite (0.06 ml; 0.28 mmol) was added dropwise. The reaction was stirred for 3 h under argon atmosphere at room temperature. The reaction mixture was diluted with CH_2Cl_2 and washed with 5 % aq $NaHCO_3$ (2 x 10 mL) and H_2O (10 mL), then the organic layer was dried over anhydrous $MgSO_4$, filtered and evaporated under reduced pressure. The crude product was purified by the flash chromatography (silica gel, petroleum ether/acetone, 2:1 *v/v*) to obtain pure product **6a** as a white solid in 92 % yield (0.16 g; 0.13 mmol). **TLC:** R_f= 0.52 ($CHCl_3$/acetone, 95:5 *v/v*).

^{31}P NMR: (176,03 MHz MHz, C_6H_6) δ: 149.89, 148.04.

2.2 Synthesis of ms²t⁶A-phosphoramidite 6b from 4b.

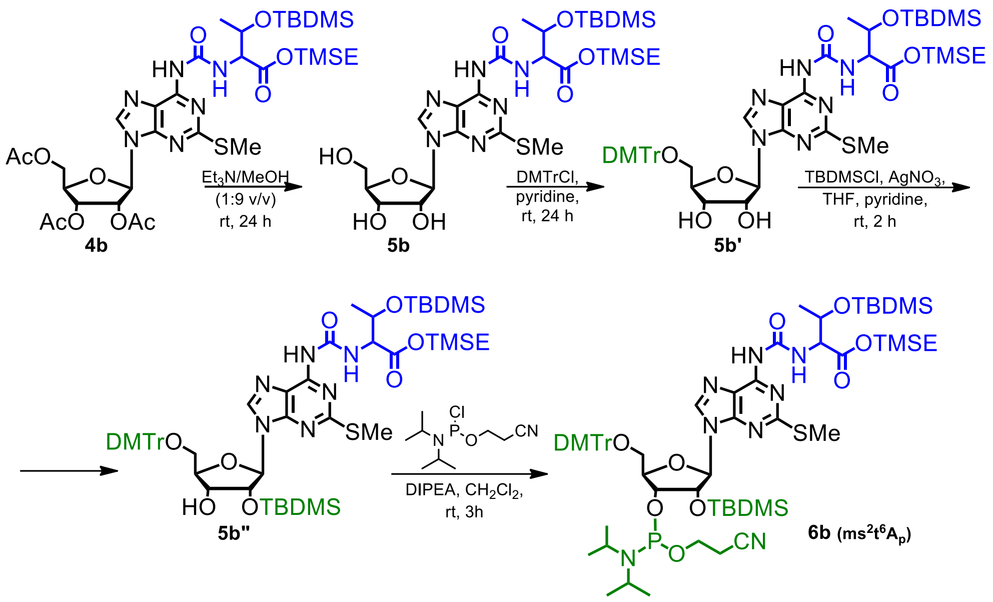

<u>*N*-[[9-(β-D-Ribofuranosyl)-2-methylthiopurin-6-yl]carbamoyl]-*O*-*tert*-butyldimethylsilyl-*L*-threonine trimethylsilylethyl ester (**5b**)</u>

Ref: K. Debiec, M. Matuszewski, K. Podskoczyj, G. Leszczyńska, E. Sochacka, *Chem. Eur. J.*, **2019**, *25*, 13309; F. Himmelsbach, B. S. Schulz, T. Trichtinger, R. Charubala, W. Pfleiderer, *Terahedron*, **1984**, *40*, 59.

To fully-protected nucleoside **4b** (156 mg, 0.20 mmol) solution of distilled solvents: TEA/MeOH (2.2 mL, 1:9 *v/v*) was added. The reaction mixture was stirred for 24 h at room temperature and the solvents were removed under reduced pressure. The oily residue was co-evaporated with toluene (2 x 5 mL). The crude product was purified by column chromatography (silica gel, 0-4 % MeOH in $CHCl_3$) to obtain pure product **5b** in 85 % yield (114 mg, 0,17 mmol). **TLC:** R_f = 0.18 ($CHCl_3$/MeOH, 95:5 *v/v*);

^{1}H NMR (700 MHz, DMSO-d_6) δ: 9.89 (s, 1H, NH-6), 9.26 (d, 1H, ^3J=8.6 Hz, NH Thr), 8.51 (s, 1H, H-8), 5.93 (d, 1H, ^3J=5.7 Hz, H-1'), 5.48 (d, 1H, ^3J=6.0 Hz, 2'OH), 5.21 (d, 1H, ^3J=5.0 Hz, 3'OH), 4.99 (t, 1H, ^3J=5.6 Hz, 5'OH), 4.63 (q, 1H, ^3J=5.6 Hz, H-2'), 4.48-4.42 (m, 2H, CH-α Thr, CH-β Thr), 4.24-4.16 (m, 2H, H-3', O-CH TMSE), 4.13-4.10 (m, 1H, O-CH TMSE), 3.94-3.66 (q, 1H, ^3J=4.2 Hz, H-4'), 3.66 (ddd, 1H, ^3J=11.9 Hz, ^3J=5.5 Hz, ^3J=4.3 Hz, H-5'), 3.56 (ddd, 1H, ^3J=11.8 Hz, ^3J=5.7 Hz, ^3J=4.4 Hz, H-5"H), 2.56 (s, 3H, S-CH_3), 1.19 (d, 3H, ^3J=6.1 Hz, CH_3 Thr), 1.04-0.91 (m, 2H, Si-CH_2 TMSE), 0.85 (s, 9H, Si-C(CH_3)$_3$ TBDMS), 0.08 (s, 3H, Si-CH_3 TBDMS), 0.03 (s, 3H, Si-CH_3 TBDMS), 0.01 (s, 9H, Si(CH_3)$_3$ TMSE).

N-[[9-(5'-*O*-(4,4'-dimethoxytrityl)-β-D-ribofuranosyl)-2-methylthiopurin-6-yl]carbamoyl]-*O*-*tert*-butyldimethylsilyl-*L*-threonine trimethylsilylethyl ester (**5b'**)

Ref: K. Onizuka, M.E. Hazemi, J.M. Thomas , L.R. Monteleone, K. Yamada, S. Imoto, P.A. Beal, F. Nagatsugi, *Bioorg Med Chem.*, **2017**, *25*, 2191; V. Boudou, J. Langridge, A. V. Aerschot, C. Hendrix, A. Millar, P. Weiss, P. Herdewijn, Helv. Chim. Acta, **2000**, *83*, 152;

To a stirred solution of free ribose nucleoside **5b** (105 mg, 0.16 mmol) in dry pyridine (1.2 mL) DMTrCl (80 mg, 0.24 mmol) was added. The reaction was stirred for 15 h at room temperature. After this time the mixture was cooled to 0°C in an ice bath and quenched with H_2O (2 mL) and stirred at 0°C for 15 min. The mixture was extracted with CH_2Cl_2 (2x 10 mL) and combined organic layers were dried over anhydrous $MgSO_4$, filtered and the solvent was evaporated. The oily residue was co-evaporated with toluene (2 x 5 mL). The crude product was purified by column chromatography (silica gel, 0-2 % MeOH in $CHCl_3$) to obtain **5b'** as a white solid in 91 % yield (143 mg, 0.14 mmol). **TLC:** R_f = 0.43 ($CHCl_3$/MeOH, 95:5 *v/v*).

^{1}**H NMR** (700 MHz, DMSO-d_6) δ: 9.91 (s, 1H, NH-6), 9.29 (d, 1H, ^3J=8.4 Hz, NH Thr), 8.42 (s, 1H, H-8), 7.35-7.27 (m, 2H, H_{Ar} DMTr), 7.23-7.18 (m, 6H, H_{Ar} DMTr), 7.18-7.15 (m, 1H, H_{Ar} DMTr), 6.81-6.77 (m, 4H, H_{Ar} DMTr), 5.98 (d, 1H, ^3J=4.2 Hz, H-1'), 5.58 (d, 1H, ^3J=5.4 Hz, 2'OH), 5.24 (d, 1H, ^3J=5.9 Hz, 3'OH), 4.77 (q, ^3J=5.3 Hz, H-2'), 4.49-4.44 (m, 2H, CH-α Thr, CH-β Thr), 4.37 (q, 1H, ^3J=5.5 Hz, H-3'), 4.22-4.18 (m, 1H, O-CH TMSE), 4.14-4.06 (m, 2H, H-4', O-CH TMSE), 3.71 (s, 3H, O-CH_3 DMTr), 3.71 (s, 3H, O-CH_3 DMTr), 3.25 (dd, 1H, ^2J=10.3 Hz, ^3J=5.9 Hz, H-5'), 3.18 (dd, 1H, ^2J=10.3 Hz, ^3J=3.4 Hz, H-5"), 2.43 (s, 3H, S-CH_3), 1.22 (d, 3H, ^3J=6.3 Hz, CH_3 Thr), 1.02-0.95 (m, 2H, Si-CH_2 TMSE), 0.86 (s, 9H, Si-C(CH_3)$_3$ TBDMS), 0.09 (s, 3H, Si-CH_3 TBDMS), 0.04 (s, 3H, Si-CH_3 TBDMS), 0.01 (s, 9H, Si(CH_3)$_3$ TMSE).

N-[[9-(2′-*O*-*tert*-butyldimethylsilyl-5′-*O*-(4,4′-dimethoxytrityl)-β-D-ribofuranosyl)-2-methylthio-purin-6-yl]carbamoyl]-*O*-*tert*-butyldimethylsilyl-*L*-threonine trimethylsilylethyl ester (**5b"**)

Ref: K. Onizuka, M.E. Hazemi, J.M. Thomas , L.R. Monteleone, K. Yamada, S. Imoto, P.A. Beal, F. Nagatsugi, *Bioorg Med Chem.*, **2017**, *25*, 2191; V. Boudou, J. Langridge, A. V. Aerschot, C. Hendrix, A. Millar, P. Weiss, P. Herdewijn, Helv. Chim. Acta, **2000**, *83*, 152;

To solution of 5′-DMTr nucleoside **5b'** (122 mg, 0.13 mmol) in freshly distilled THF (0.50 mL), anhydrous pyridine (50 μL, 0.65 mmol) and $AgNO_3$ (32 mg, 0.19 mmol) were added. The reaction mixture was stirred at room temperature for 30 min in darkness. Then TBDMS-Cl (34 mg, 0.23 mmol) was added and stirring was continued at room temperature for 2 h. After this time, the reaction mixture was diluted with CH_2Cl_2 (5 mL), filtered through Celite and washed with CH_2Cl_2. The solution was extracted with saturated $NaHCO_3$ (2 x 5 mL) and brine (5 mL). The organic layer

was dried over $MgSO_4$, filtered and concentrated under reduced pressure. The oily residue was co-evaporated with toluene (2 x 5 mL) and the crude product was purified by the column chromatography (silica gel, 0-8 % acetone in $CHCl_3$). The pure 2'-isomer **5b"** was obtained as a white solid in 57 % yield (80 mg, 0.074 mmol). The 3'-regioisomer (38 mg, 0.035 mmol) was dissolved in methanol with few drops of TEA, to give equimolar mixture of 2' and 3' TBDMS isomers from which 2'-TBDMS nucleoside was chromatographically isolated to give finally 99 mg of **5b"** (0.091 mmol; 70 % yield). **TLC:** R_f = 0.42 ($CHCl_3$/MeOH, 98:2 *v/v*).

^{1}H NMR (700 MHz, DMSO-d_6) δ: 9.94 (s, 1H, NH-6), 9.27 (d, 1H, ^3J=8.6 Hz, NH Thr), 8.41 (s, 1H, H-8), 7.36-7.32 (m, 2H, H_{Ar} DMTr), 7.26-7.21 (m, 6H, H_{Ar} DMTr), 7.21-7.16 (m, 1H, H_{Ar} DMTr), 6.85-6.80 (m, 4H, H_{Ar} DMTr), 5.99 (d, 1H, ^3J=4.5 Hz, H-1'), 5.19 (d, 1H, ^3J=6.1 Hz, 3'OH), 4.89 (t, 1H, ^3J=4.7 Hz, H-2'), 4.48-4.42 (m, 2H, CH-α Thr, CH-β Thr), 4.30 (q, 1H, ^3J=5.6 Hz, H-3'), 4.20-4.18 (m, 1H, O-CH TMSE), 4.15-4.06 (m, 2H, H-4', O-CH TMSE), 3.72 (s, 3H, O-CH_3 DMTr), 3.71 (s, 3H, O-CH_3 DMTr), 3.31-3.30 (m, 1H, H-5'), 3.23 (dd, 1H, ^2J=10.3 Mz, ^3J=3.4 Hz, H-5"), 2.39 (s, 3H, S-CH_3), 1.21 (d, 3H, ^3J=6.2 Hz, CH_3 Thr), 0.99-0.95 (m, 2H, Si-CH_2 TMSE), 0.85 (s, 9H, Si-$C(CH_3)_3$ TBDMS), 0.77 (s, 9H, Si-$C(CH_3)_3$ TBDMS), 0.08 (s, 3H, Si-CH_3 TBDMS), 0.03 (s, 3H, Si-CH_3 TBDMS), -0.00 (s, 9H, $Si(CH_3)_3$ TMSE), -0.02 (s, 3H, Si-CH_3 TBDMS), -0.11 (s, 3H, Si-CH_3 TBDMS).

N-[[9-(2'-*O*-*tert*-butyldimethylsilyl-3'-(2-cyanoethyl-*N*,*N*-diisopropylphosphoramidite)-5'-*O*-(4,4'-dimethoxytrityl)-β-D-ribofuranosyl)-2-methylthiopurin-6-yl]carbamoyl]-*O*-*tert*-butyldimethylsilyl-*L*-threonine trimethylsilylethyl ester (**6b**)

Ref: V. Boudou, J. Langridge, A. V. Aerschot, C. Hendrix, A. Millar, P. Weiss, P. Herdewijn, Helv. Chim. Acta, **2000**, *83*, 152;

To stirred solution of 5'-DMTr, 2'-TBDMS-ms^{2}t^{6}A **5b"** (80 mg, 0.08 mmol) in freshly distilled CH_2Cl_2 (0.4 mL) under argon atmosphere anhydrous DIPEA was added (0.05 mL, 0.29 mmol). Then 2-cyanoethyl-*N,N*-diisopropyl chlorophosphoramidite (0.04 ml; 0.14 mmol) were added dropwise. The reaction was stirred for 3 h under argon atmosphere at room temperature. The reaction mixture was diluted with CH_2Cl_2 (5 mL) and washed with 5 % aq $NaHCO_3$ (2 x 5 mL) and H_2O (5 mL), then the organic layer was dried over anhydrous $MgSO_4$, filtered and evaporated under reduced pressure. The crude product was purified by the flash chromatography (silica gel, petroleum ether/acetone, 2:1 v/v) to obtain pure product **6b** as a white solid in 90 % yield (85 mg; 0.07 mmol). **TLC:** R_f= 0.53 ($CHCl_3$/acetone, 95:5 *v/v*).

^{31}P NMR: (283 MHz, C_6H_6,) δ: 150.90, 149.49.

III. Spectroscopic data.

3.1 NMR and IR spectra of threonine derivatives.

Figure S1 ^{1}H NMR spectrum of *N*-(t*ert*-butoxycarbonyl)-*O*-*tert*-butyldimethylsilyl-L-threonine trimethylsilylethyl ester (**1**)

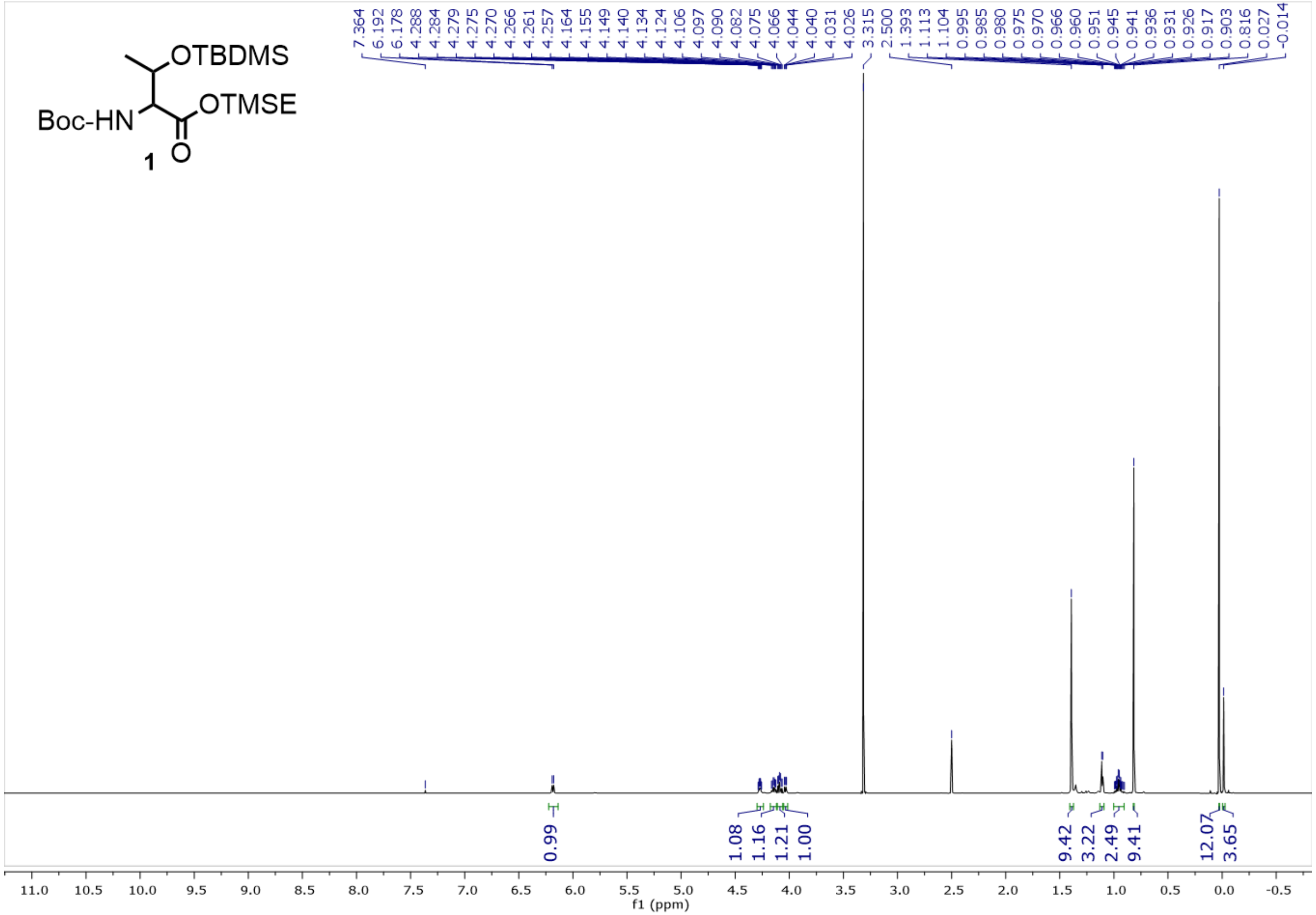

^{1}H NMR (700 MHz, DMSO-d_6) δ: 6.19 (d, 1H, ^3J=9.4 Hz, NH), 4.27 (dq, 1H, ^3J=6.2 Hz, ^3J=3.1 Hz, CH-β), 4.14 (td, 1H, ^3J=10.7 Hz, ^3J=6.5 Hz, O-CH TMSE), 4.09 (td, 1H, ^3J=10.7 Hz, ^3J=6.2 Hz, O-CH TMSE), 4.04 (dd, 1H, ^3J=9.4 Hz, ^3J=3.1 Hz, CH-α), 1.39 (s, 9H, C-$(CH_3)_3$ Boc), 1.11 (d, 3H, ^3J=6.2 Hz, CH_3), 1.01-0.89 (m, 2H, Si-CH_2 TMSE), 0.82 (s, 9H, Si-C$(CH_3)_3$ TBDMS), 0.03 (s, 12H, Si$(CH_3)_3$ TMSE; Si-CH_3 TBDMS), -0.01 (s, 3H, Si-CH_3 TBDMS).

Figure S2 ^{13}C NMR spectrum of *N*-(*tert*-butoxycarbonyl)-*O*-*tert*-butyldimethylsilyl-L-threonine trimethylsilylethyl ester (**1**)

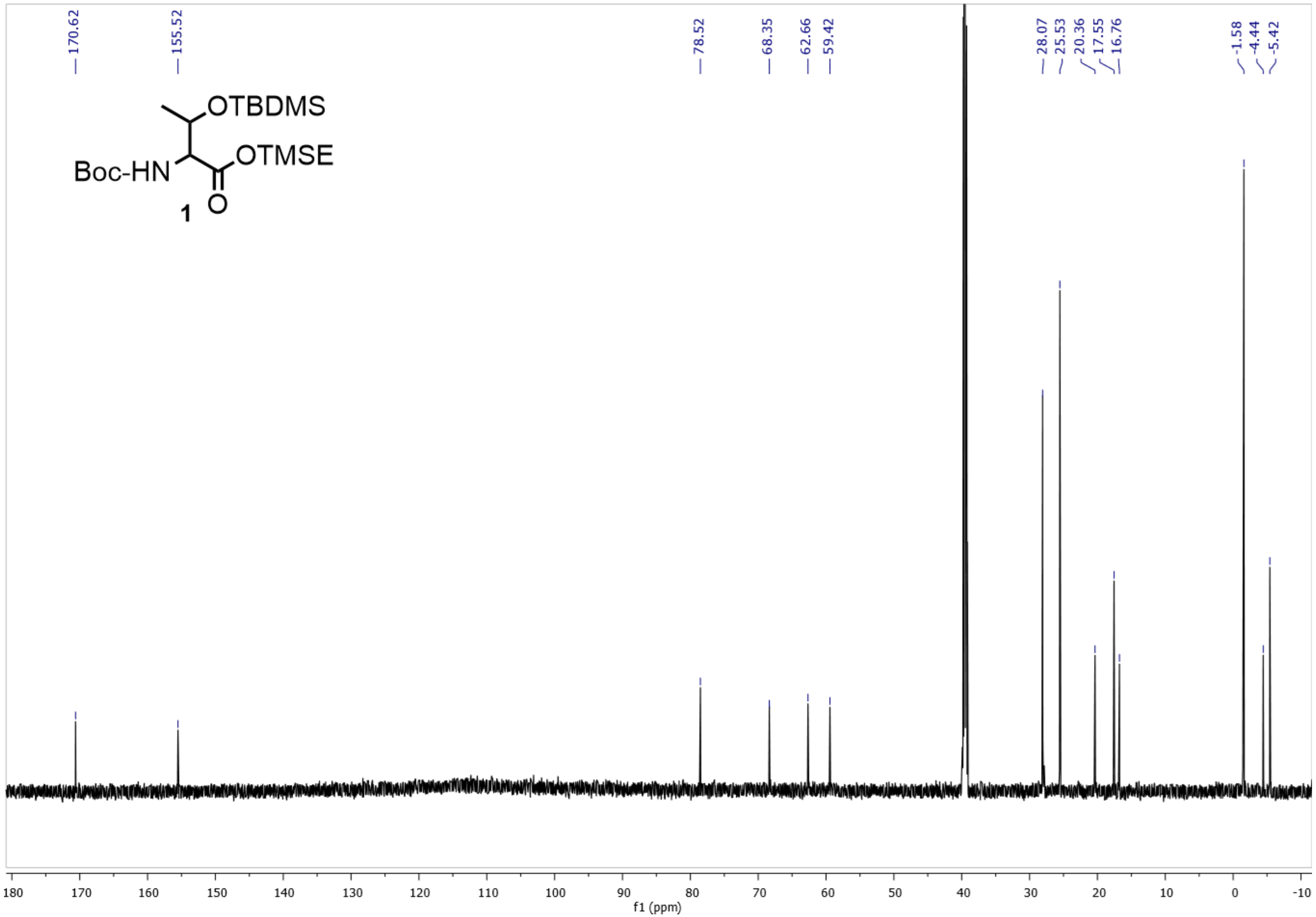

^{13}C NMR: (176,03 MHz DMSO-d$_6$) δ: 170.62 (C=O), 155.52 (C=O), 78.52 (C-(CH$_3$)$_3$ Boc), 68.35 (CH-β), 62.66 (O-CH$_2$ TMSE), 59.42 (CH-α), 28.07 (C-(CH$_3$)$_3$ Boc), 25.53 (Si-C-CH$_3$)$_3$ TBDMS), 20.36 (CH$_3$), 17.55 (Si-C-CH$_3$)$_3$ TBDMS), 16.76 (Si-CH$_2$ TMSE), -1.58 (Si(CH$_3$)$_3$ TMSE), -4.44 (Si-CH$_3$ TBDMS), -5.42 (Si-CH$_3$ TBDMS).

Figure S3 ^{1}H NMR spectrum of 2-(S)-isocyane-3-(*O*-*tert*-butyldimethylsilyl)-3-hydroxybutanoic 2-trimethylsilylethyl ester (**2**)

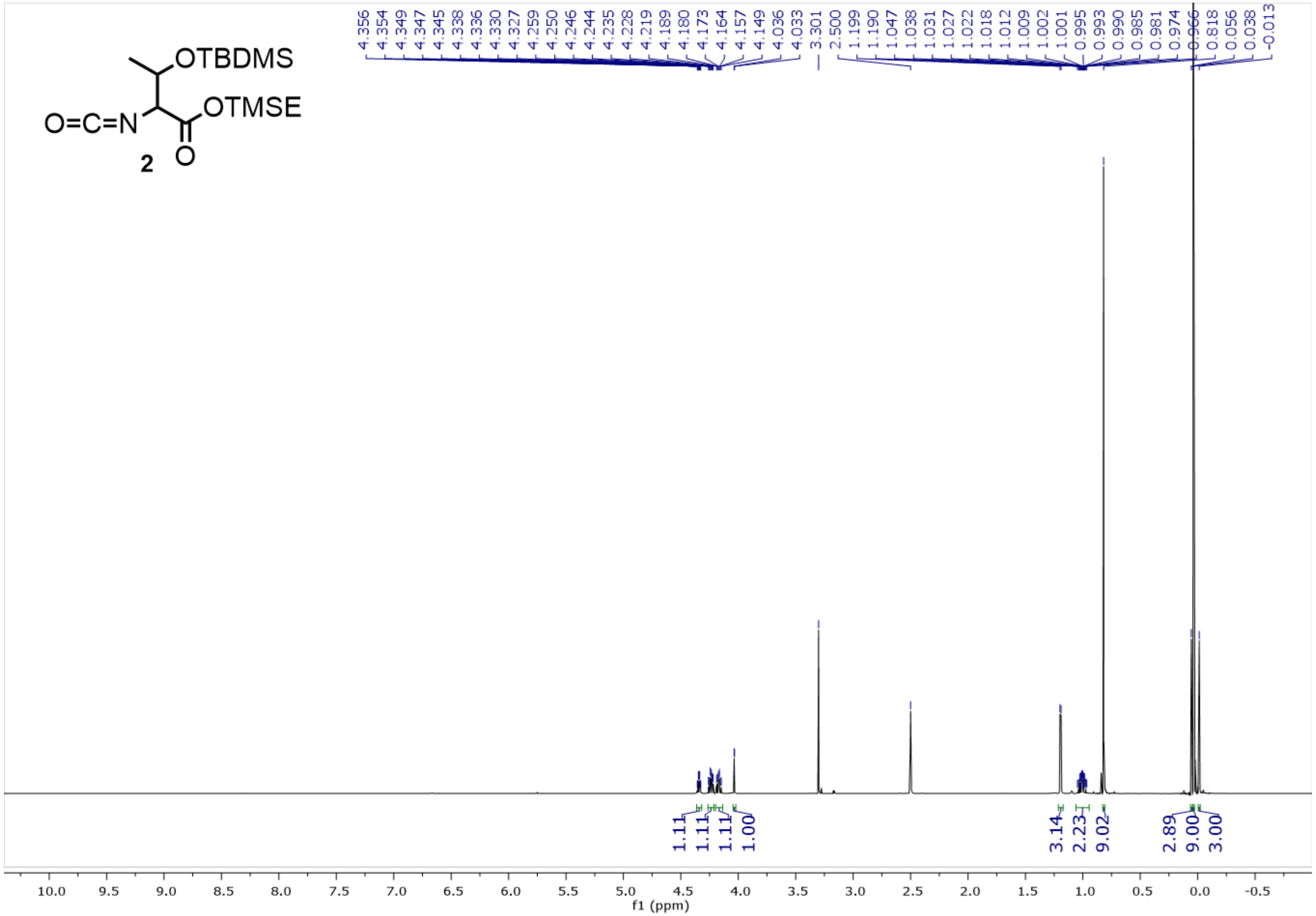

^{1}H NMR (700 MHz, DMSO-d_6) δ: 4.34 (qd, 1H, ^{3}J=6.2 Hz, ^{3}J=1.7 Hz, CH-β), 4.24 (td, 1H, ^{3}J=10.9 Hz, ^{3}J=6.4 Hz, O-CH TMSE), 4.17 (td, 1H, ^{3}J=11.1 Hz, ^{3}J=5.9 Hz, O-CH TMSE), 4.03 (d, 1H, ^{3}J=1.7 Hz, CH-α), 1.19 (d, 3H, ^{3}J=6.3 Hz, CH_3), 1.07–0.94 (m, 2H, Si-CH_2 TMSE), 0.82 (s, 9H, Si-C-$(CH_3)_3$ TBDMS), 0.06 (s, 3H, Si-CH_3 TBDMS), 0.04 (s, 9H, Si-$(CH_3)_3$ TMSE), -0.01 (s, 3H, Si-CH_3 TBDMS).

Figure S4 ^{13}C NMR spectrum of 2-(S)-isocyane-3-(*O-tert*-butyldimethylsilyl)-3-hydroxybutanoic 2-trimethylsilylethyl ester (**2**)

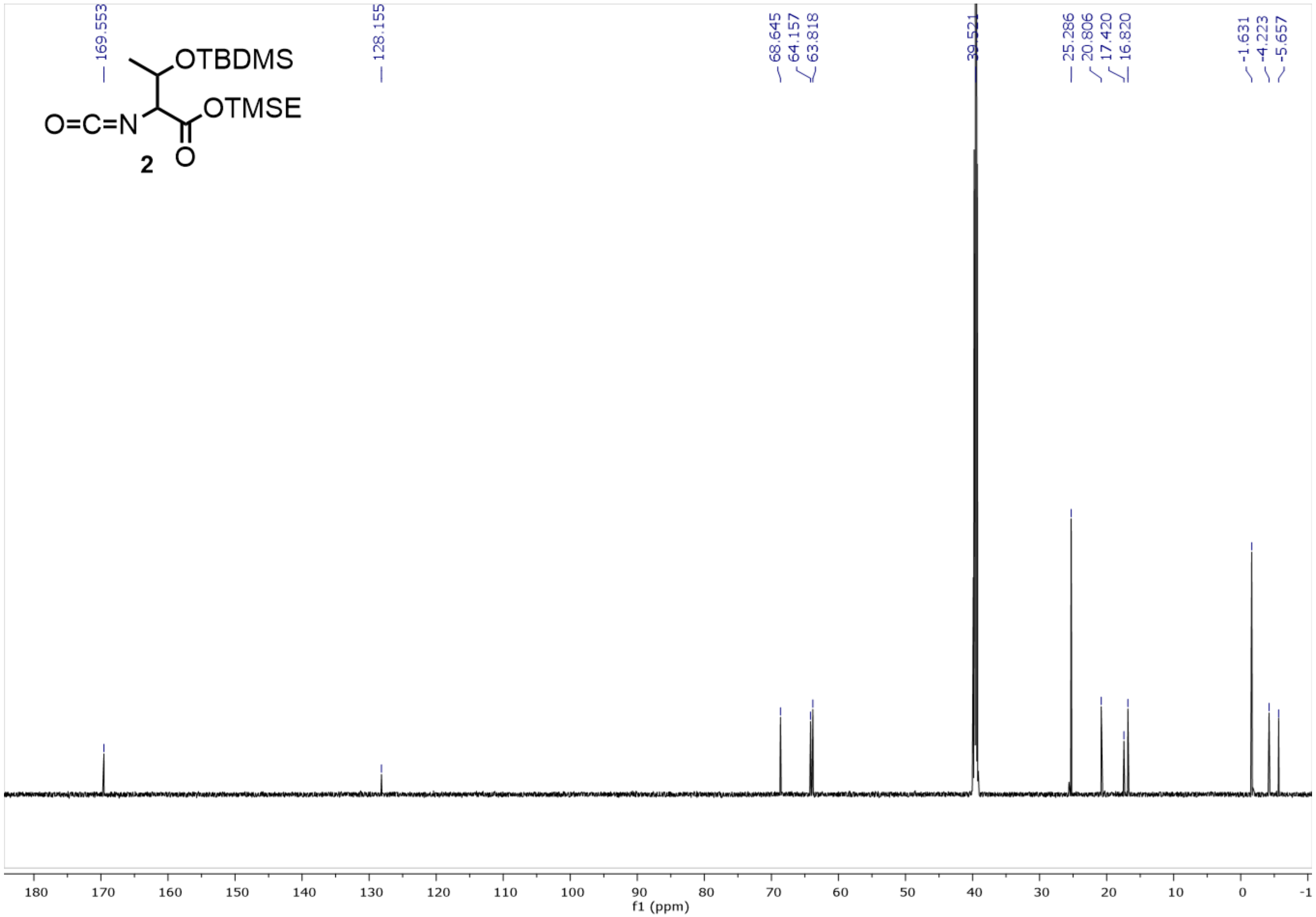

^{13}C NMR (176,03 MHz DMSO-d_6) δ: 169.55 (C=O), 128.15 (N=C=O), 68.64 (CH-β), 64.16 (O-CH_2 TMSE), 63.82 (CH-α), 25.29 (Si-C(<u>C</u>H_3)$_3$ TBDMS), 20.81 (CH_3), 17.42 (Si-<u>C</u>(CH_3)$_3$ TBDMS), 16.82 (Si-CH_2 TMSE), -1.63 (Si(CH_3)$_3$ TMSE), -4.22 (Si-CH_3 TBDMS), -5.66 (Si-CH_3 TBDMS).

Figure S5 IR spectrum of *N*-(*tert*-butoxycarbonyl)-*O*-*tert*-butyldimethylsilyl-ʟ-threonine trimethylsilylethyl ester (**1**)

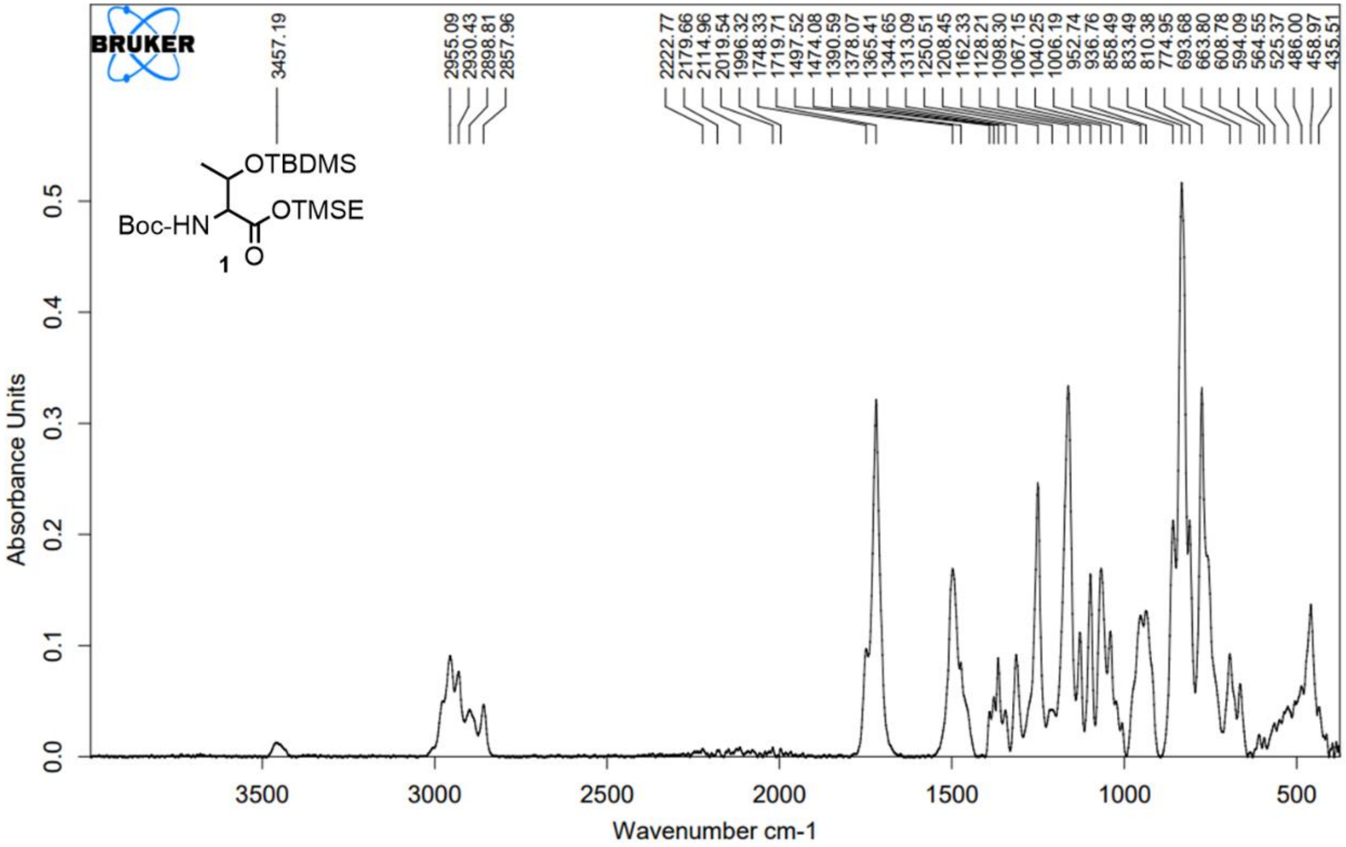

Figure S6 IR spectrum of 2-(S)-isocyane-3-(*O*-*tert*-butyldimethylsilyl)-3-hydroxybutanoic 2-trimethylsilylethyl ester (**2**)

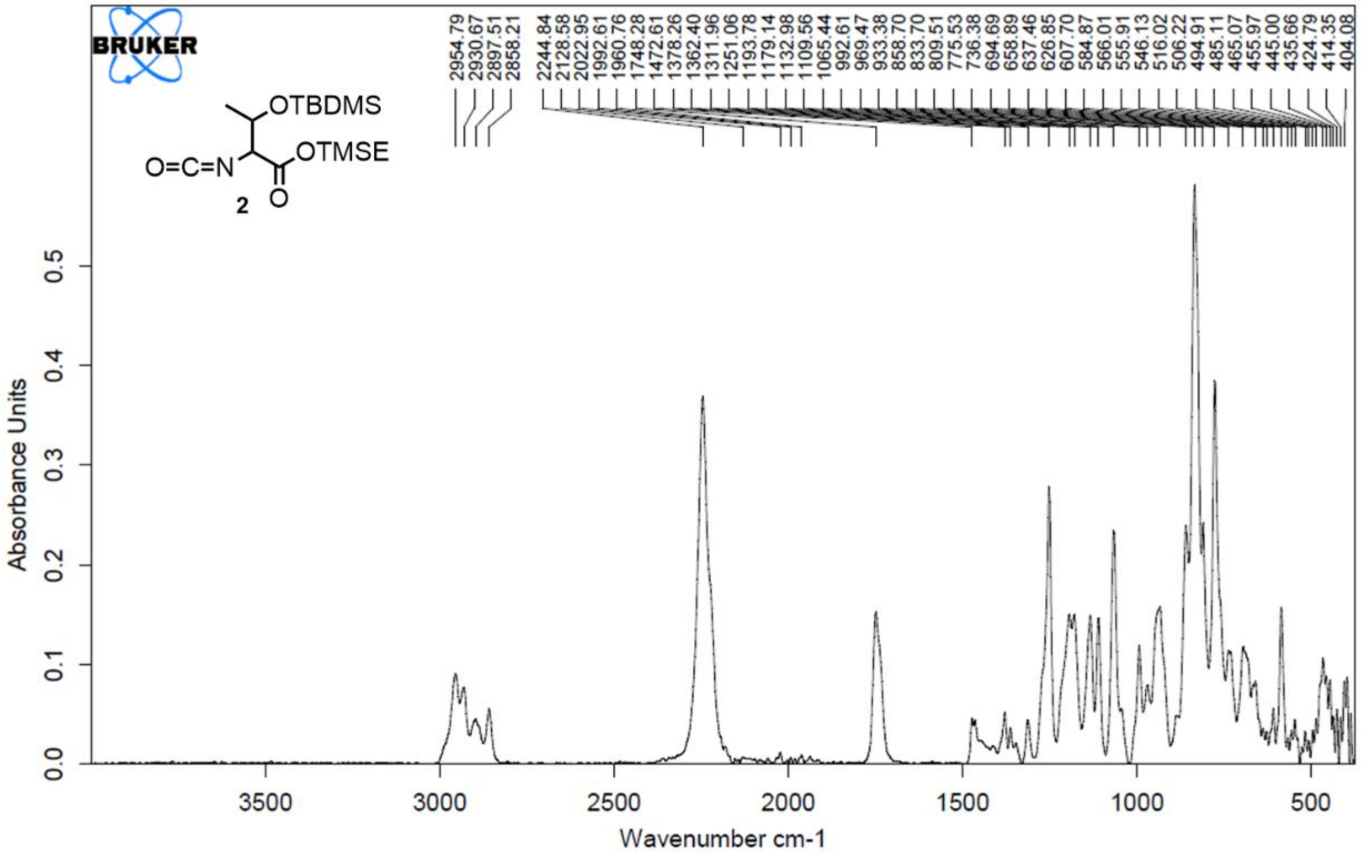

3.2 NMR spectra of adenosine derivatives

Figure S7 ^{1}H NMR spectrum of **3a**

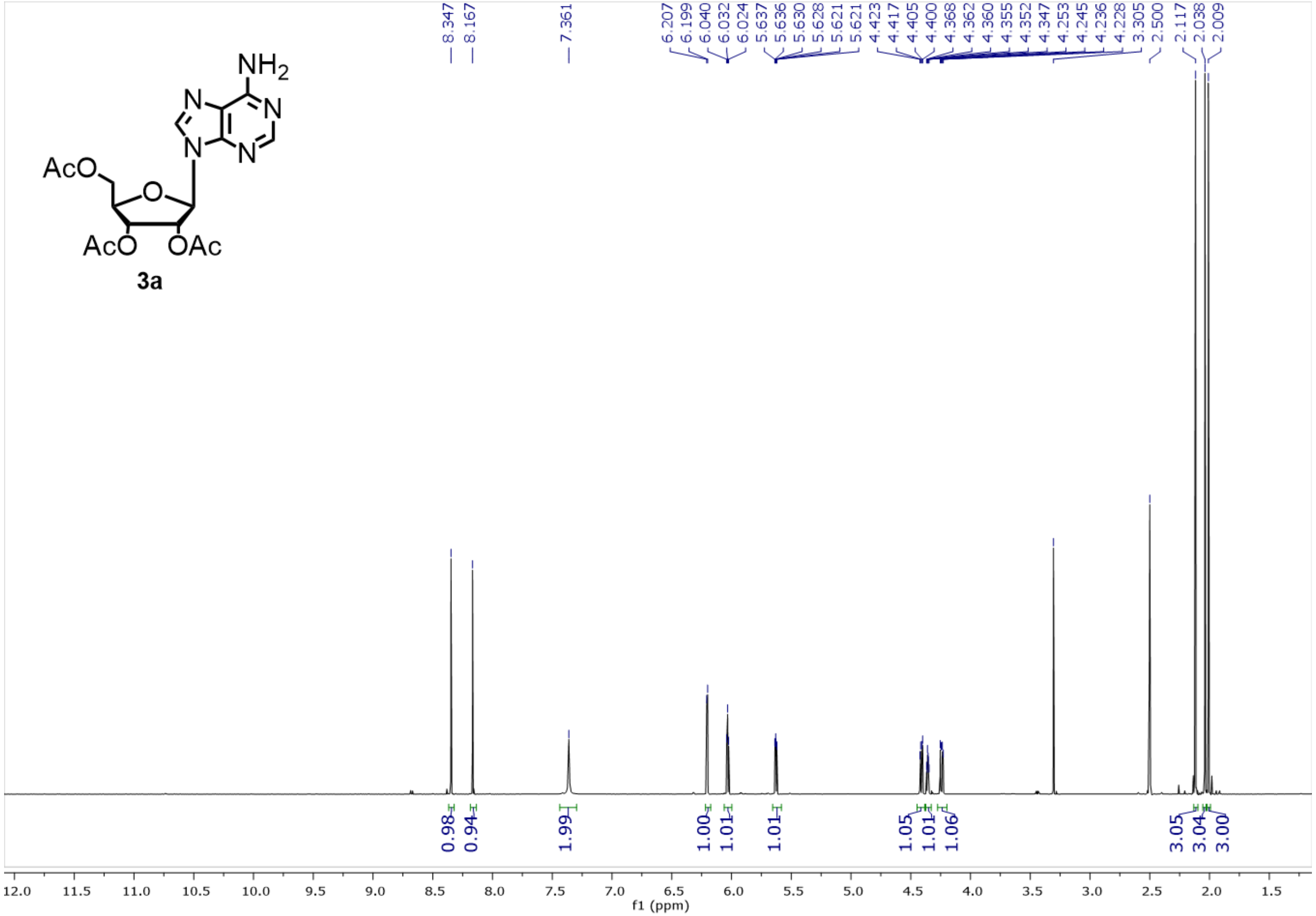

^{1}H NMR: (700 MHz, DMSO-d_6) δ: 8.35 (s, 1H, H-8), 8.17 (s, 1H, H-2), 7.36 (br s, 2H, NH_2), 6.20 (d, 1H, ^3J=5.5 Hz, H-1'), 6.03 (t, 1H, ^3J=5.7 Hz, H-2'), 5.63 (dd, 1H, ^3J=5.8 Hz, ^3J=5.1 Hz, H-3'), 4.41 (dd, 1H, ^2J=11.9 Hz, ^3J=3.8 Hz, H-5'), 4.38-4.34 (m, 1H, H-4'), 4.24 (dd, 1H, ^2J=11.9 Hz, ^3J=5.6 Hz, H-5''), 2.12 (s, 3H, CH_3-CO Ac), 2.04 (s, 3H, CH_3-CO Ac), 2.01 (s, 3H, CH_3-CO Ac).

Figure S8 ¹H NMR spectrum of **4a**

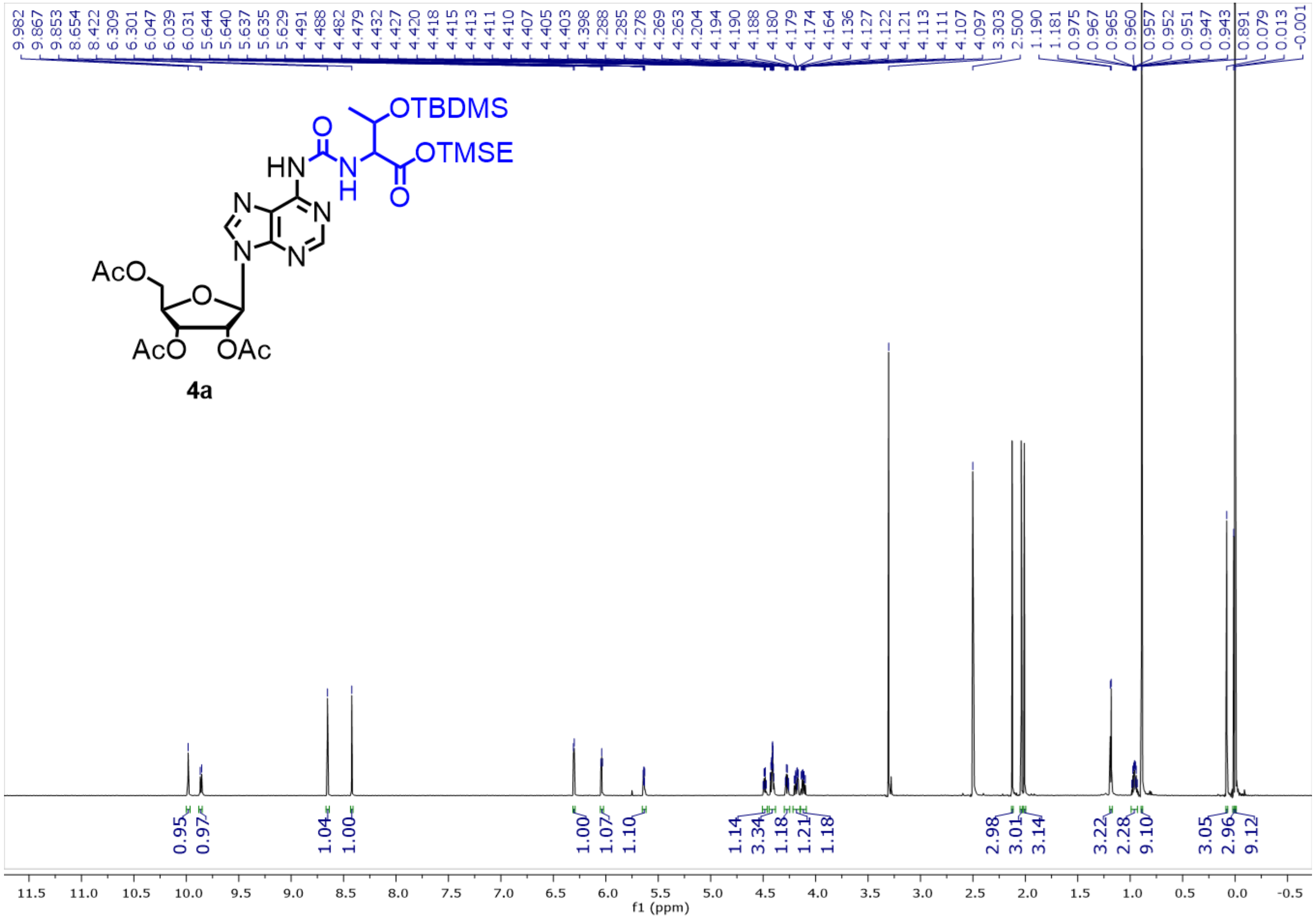

Figure S9 ¹H NMR spectrum of **5a**

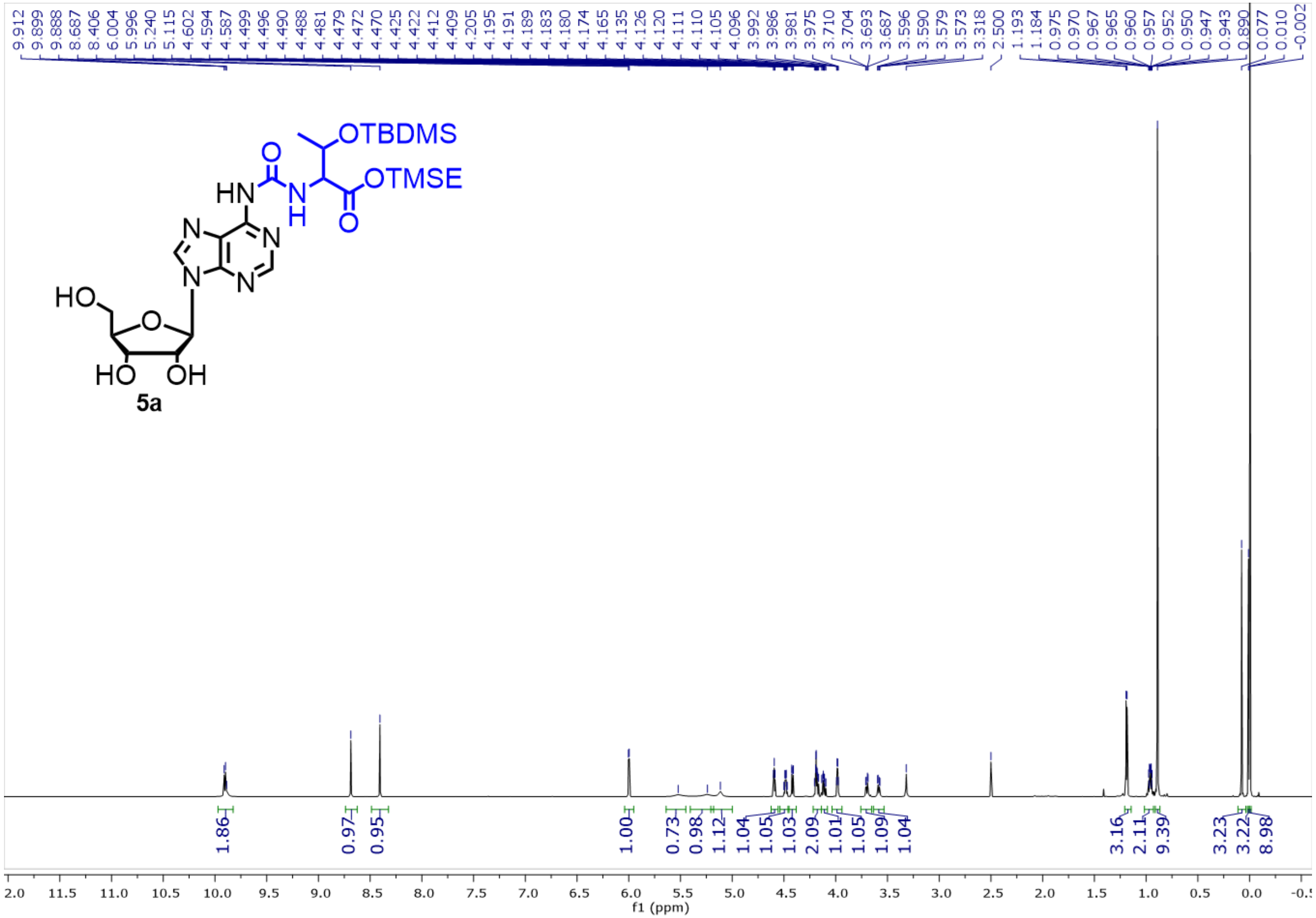

Figure S10 ^{1}H NMR spectrum of **5a’**

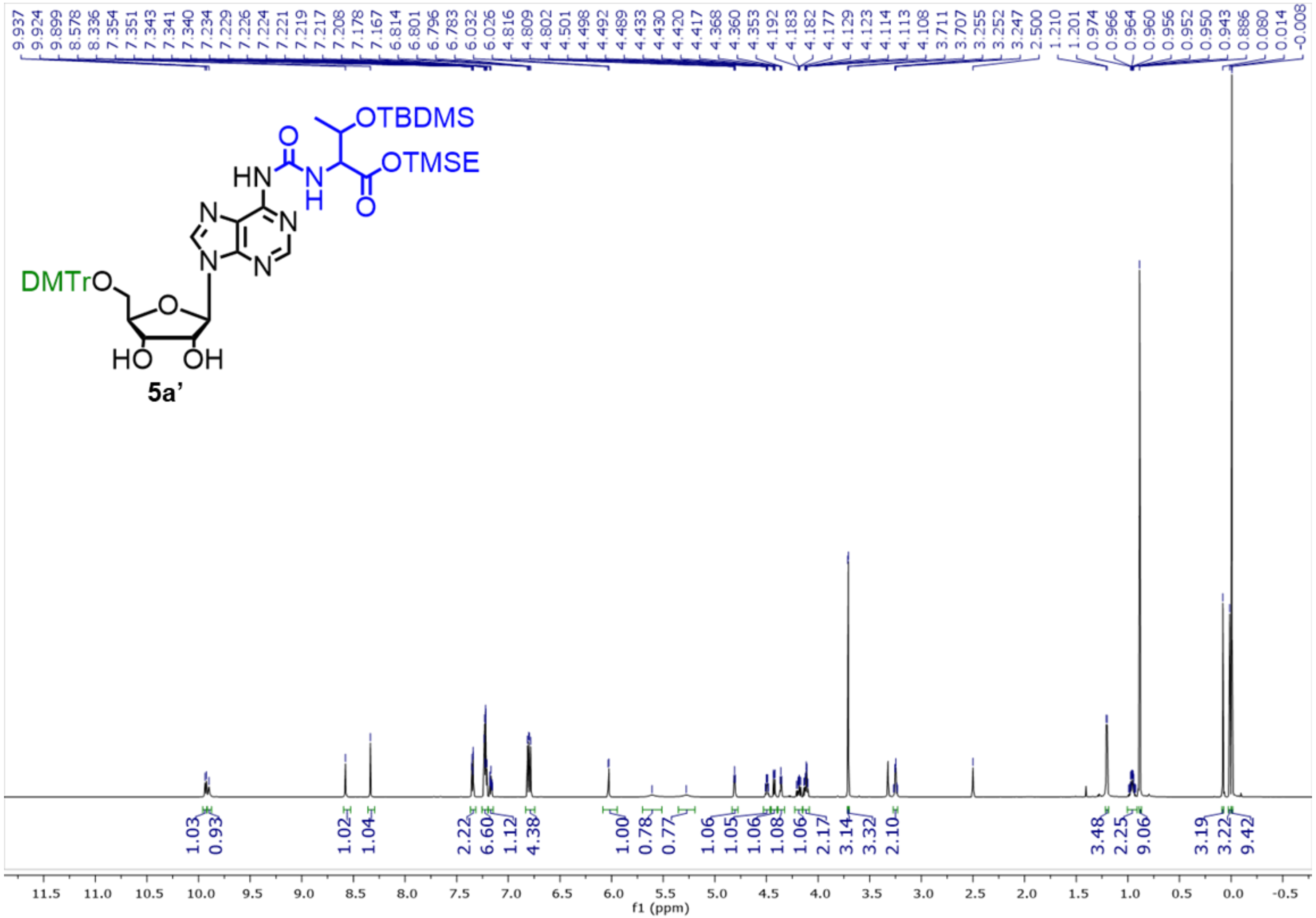

Figure S11 ^{1}H NMR spectrum of **5a”**

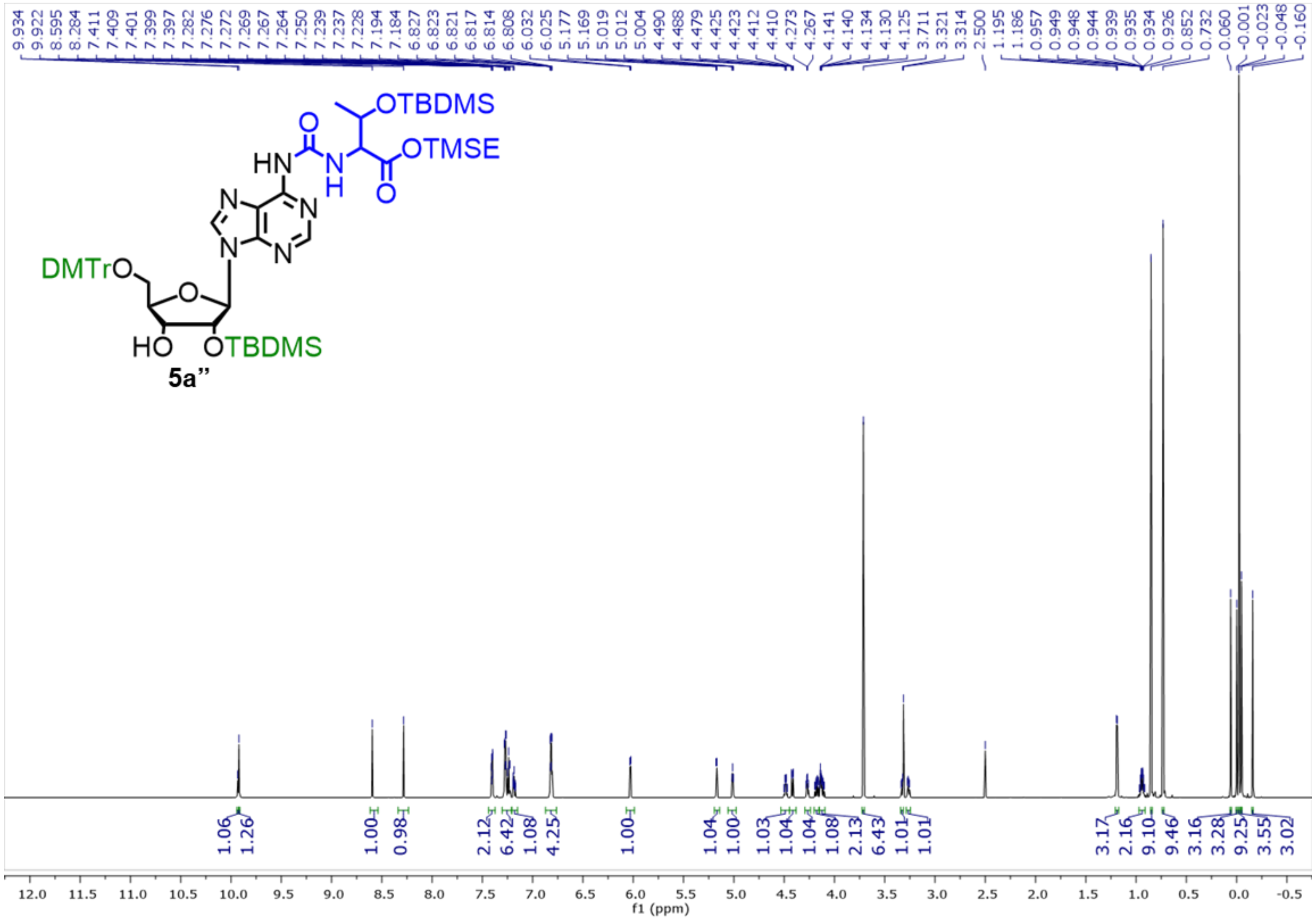

Figure S12 ^{31}P NMR spectrum of **6a**

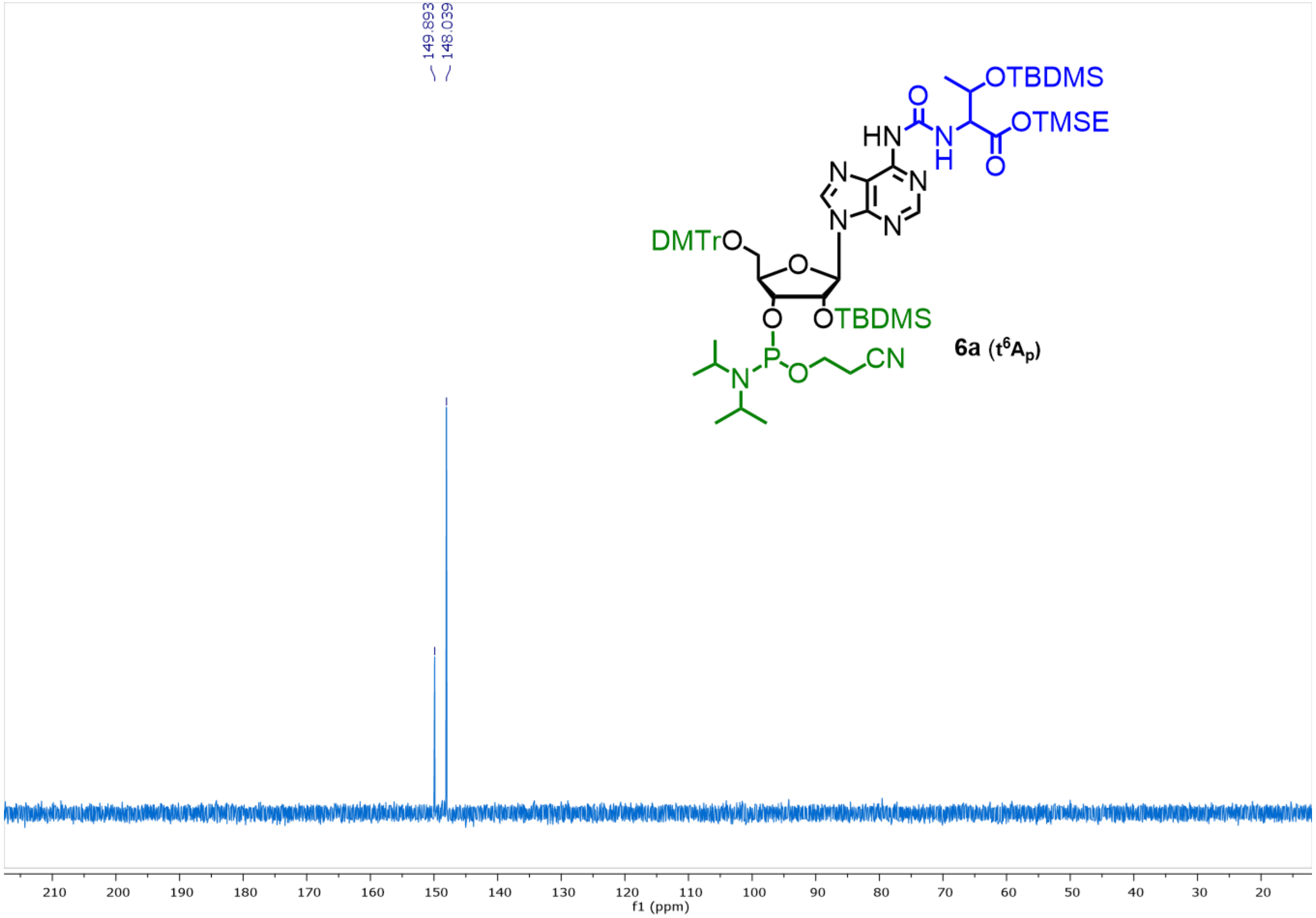

Figure S13 ^{1}H NMR spectrum of **3b**

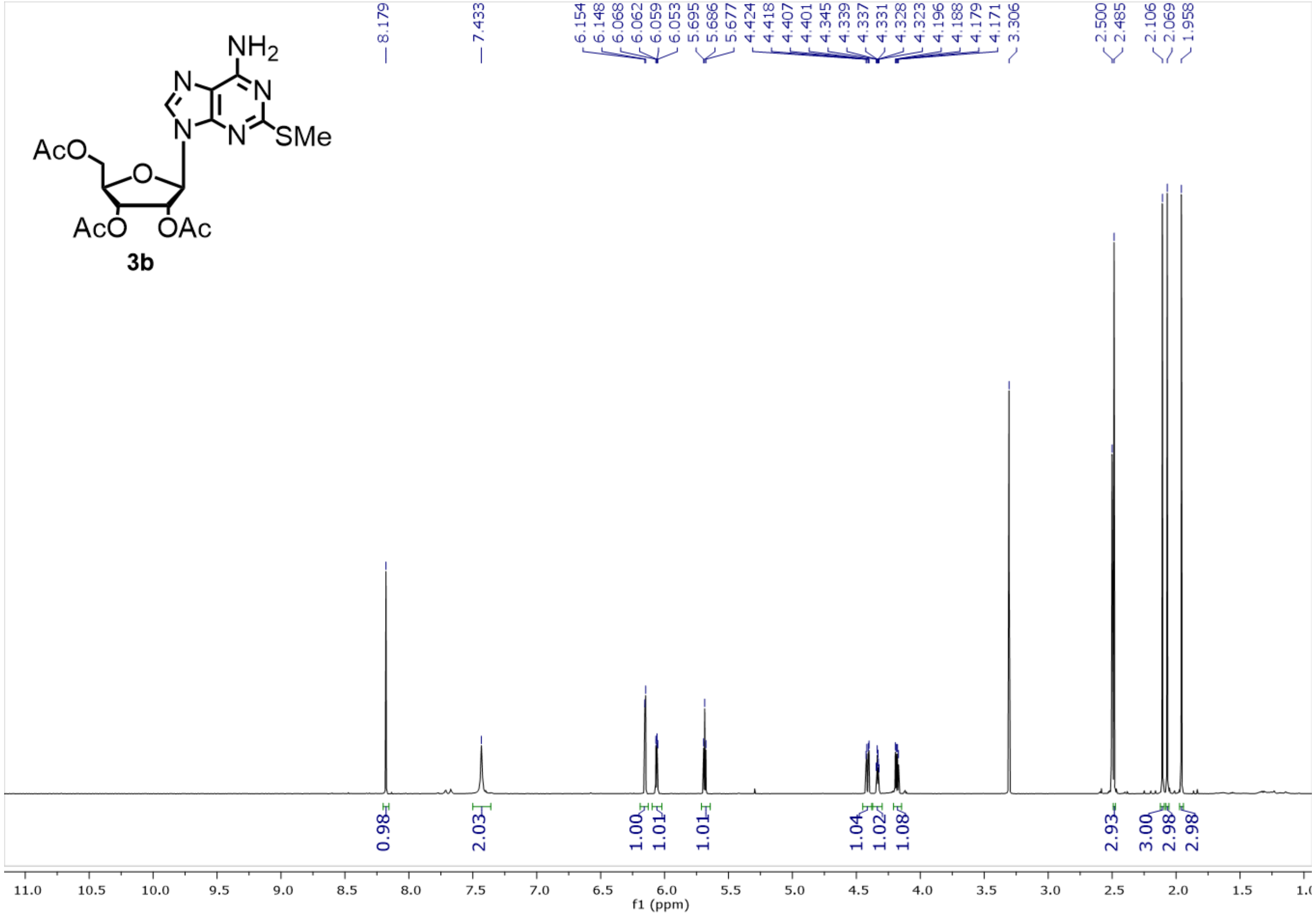

^{1}H NMR: (700 MHz, DMSO-d$_6$) δ: 8.18 (s, 1H, H-2), 7.43 (br s, 2H, NH_2), 6.15 (d, 1H, ^3J=4.3 Hz, H-1'), 6.06 (dd, 1H, ^3J=6.1 Hz,^3J=4.3 Hz, H-2'), 5.69 (t, 1H, ^3J=6.1 Hz, H-3'), 4.41 (dd, 1H, ^2J=12.0 Hz, ^3J=3.8 Hz, H-5'), 4.41 (td, 1H,^3J=5.8 Hz,^3J=3.7 Hz, H-4'), 4.18 (dd, 1H, ^2J=12.0 Hz, ^3J=5.6 Hz, H-5''), 2.48 (s, 3H, -SCH_3), 2.11 (s, 3H, CH_3-CO Ac), 2.07 (s, 3H, CH_3-CO Ac), 1.96 (s, 3H, CH_3-CO Ac).

Figure S14 ^{1}H NMR spectrum of **4b**

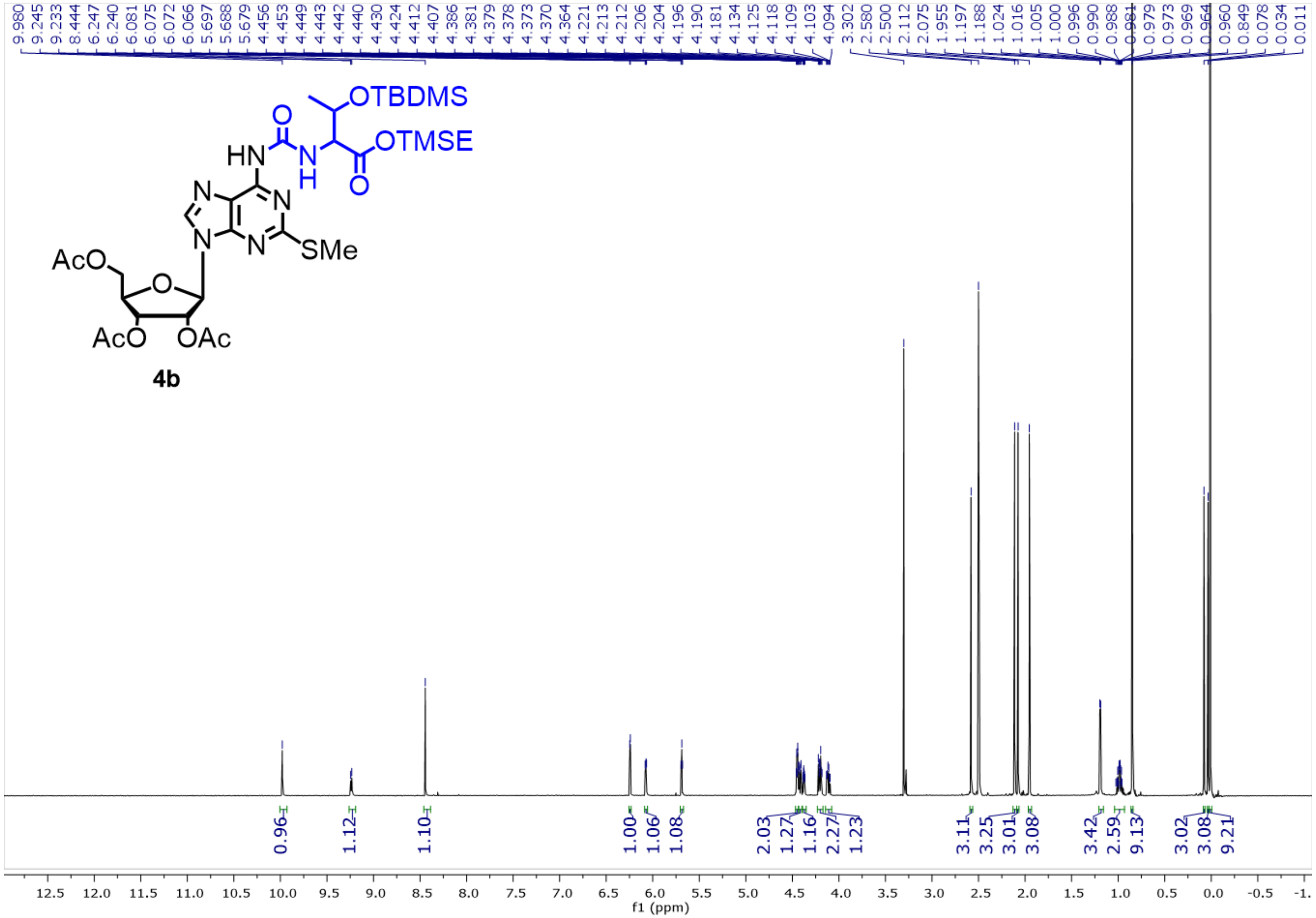

Figure S15 ^{1}H NMR spectrum of **5b**

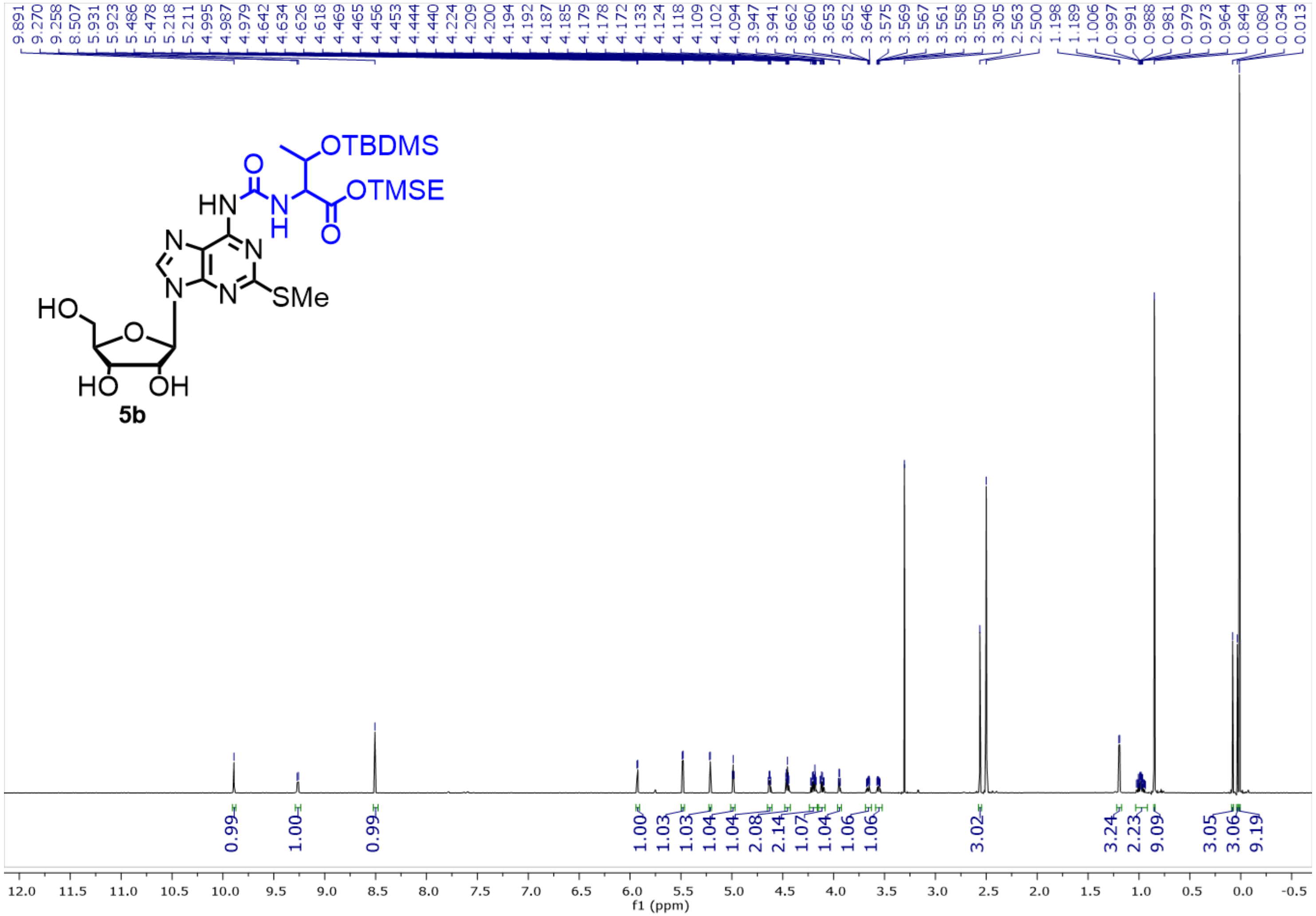

Figure S16 ^{1}H NMR spectrum of **5b’**

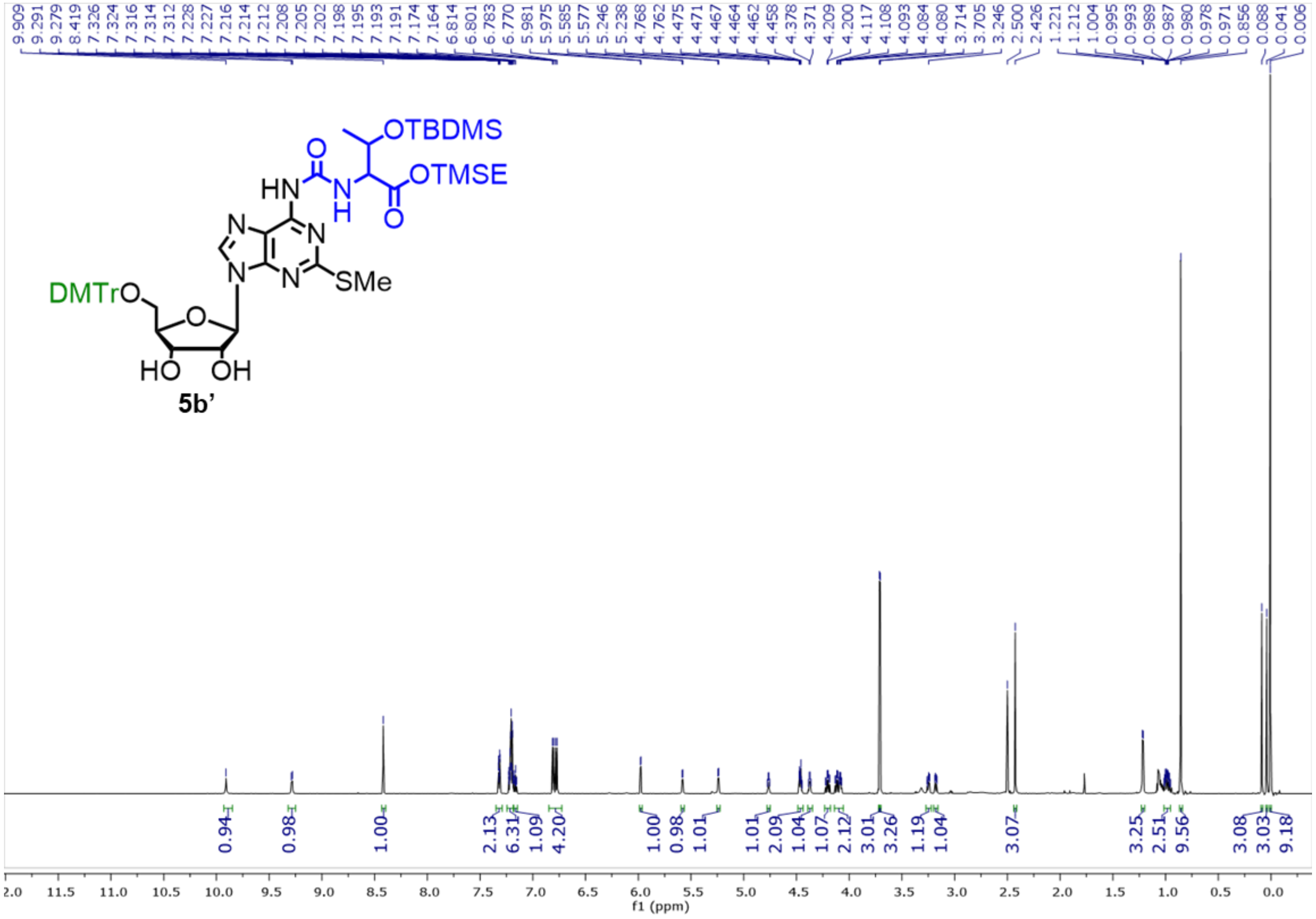

Figure S17 ^{1}H NMR spectrum of **5b"**

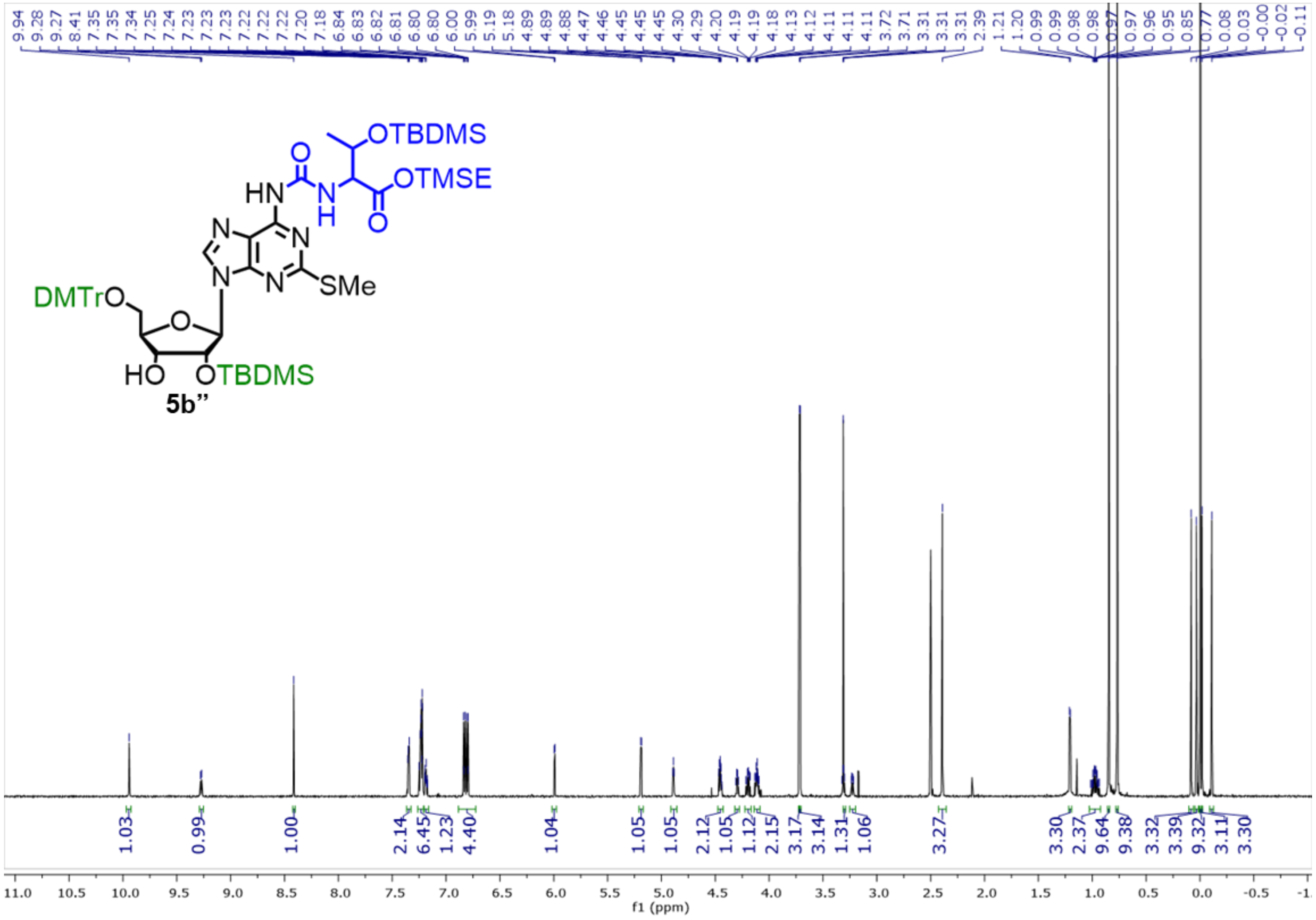

Figure S18 ^{31}P NMR spectrum of **6b**

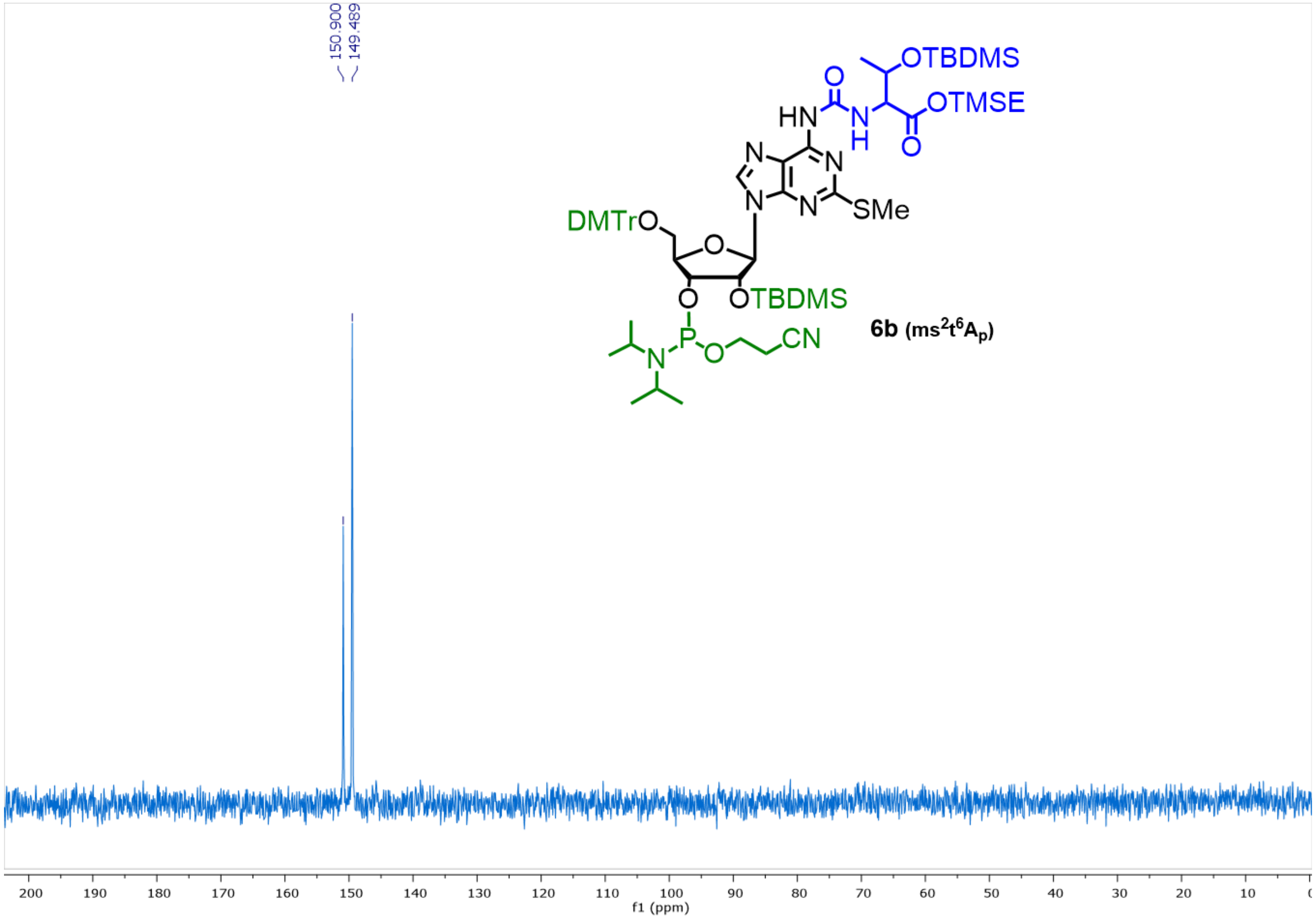

Figure S19 ^{1}H NMR spectrum of **9**

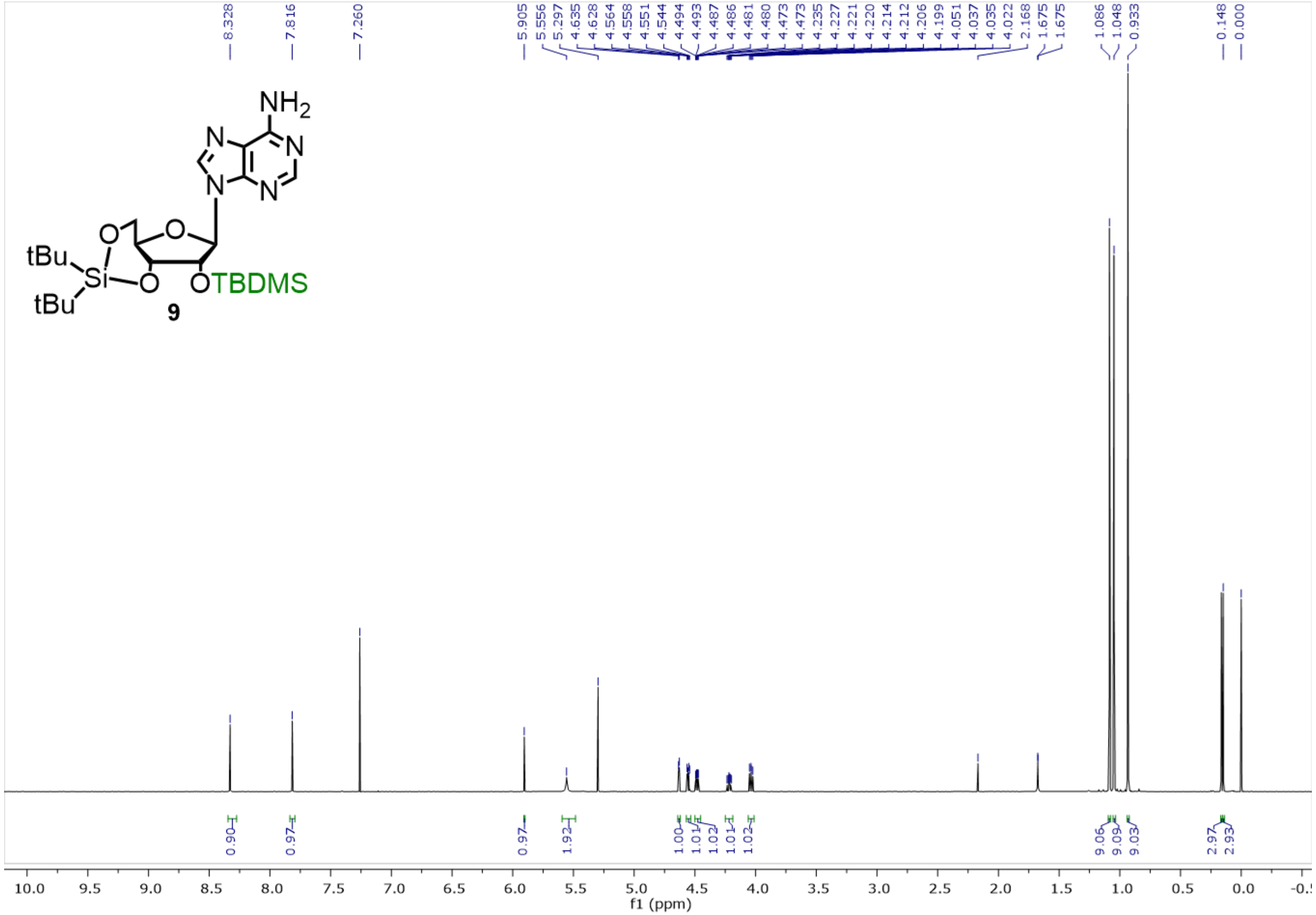

^{1}H NMR (700 MHz, $CDCl_3$) δ: 8.33 (s, 1H H-8), 7.82 (s, 1H, H-2), 5.91 (br s, 1H, H-1'), 5.56 (br s, 2H, NH_2), 4.63 (br d, 1H, ^3J=4.7 Hz, H-2'), 4.55 (dd, 1H, ^3J= 9.7 Hz, ^3J=4.7 Hz, H-3'), 4.48 (dd, 1H, ^2J= 9.3 Hz, ^3J=5.2 Hz, H-5'), 4.22 (ddd, 1H, ^3J=10.7 Hz, ^3J=9.6 Hz, ^3J=5.2 Hz, H-4'), 4.04 (dd, 1H, ^3J=10.6 Hz, ^2J=9.2 Hz, H-5''), 1.09 (s, 9H, Si-$C(CH_3)_3$ tBu_2Si), 1.05 (s, 9H, Si-$C(CH_3)_3$ tBu_2Si), 0.93 (s, 9H, Si-$C(CH_3)_3$ TBDMS), 0.16 (s, 3H, Si-CH_3 TBDMS), 0.15 (s, 3H, Si-CH_3 TBDMS).

Figure S20 ^{1}H NMR spectrum of **10**

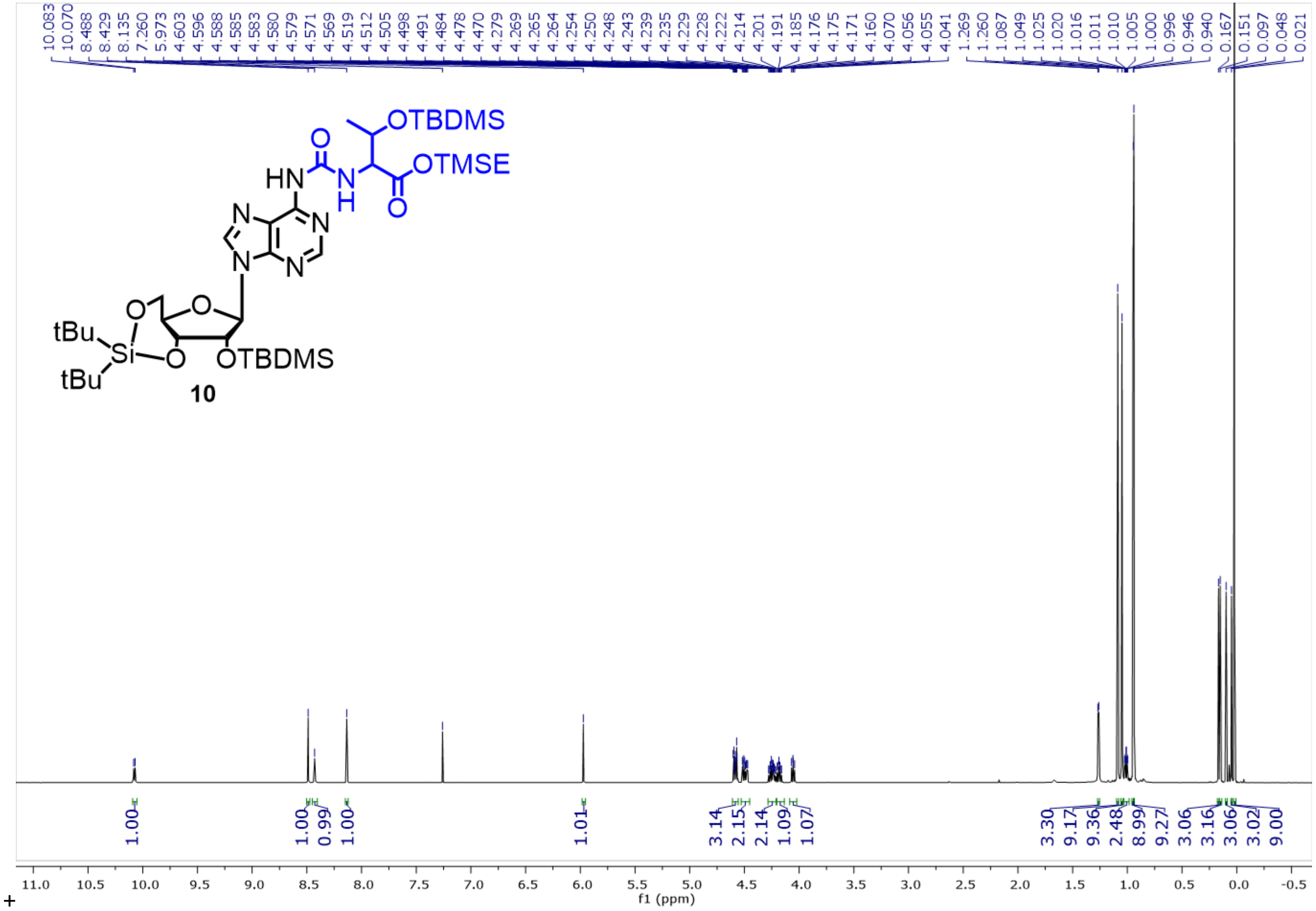

Figure S21 ^{13}C NMR spectrum of **10**

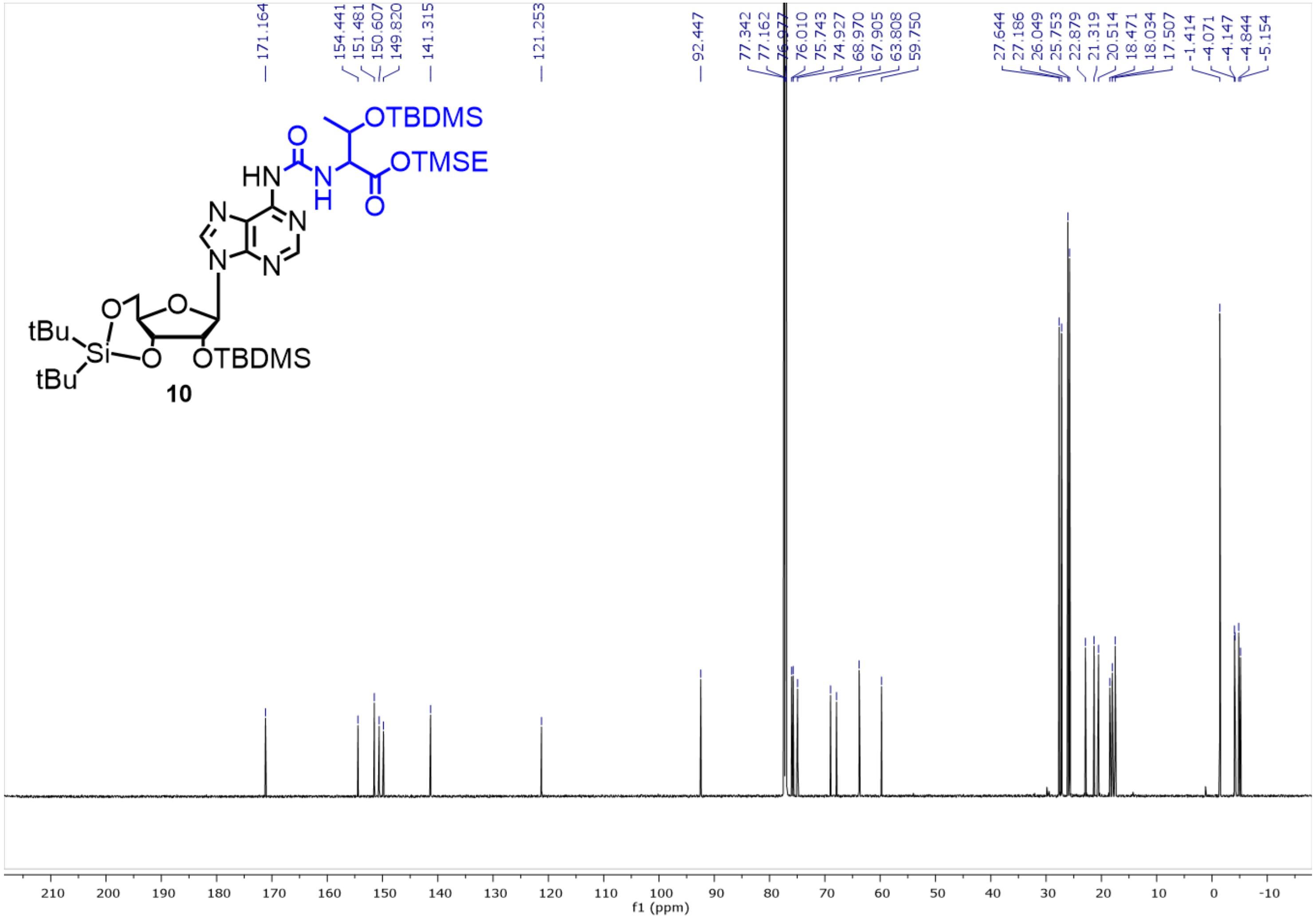

Figure S22 ^{1}H NMR spectrum of **11**

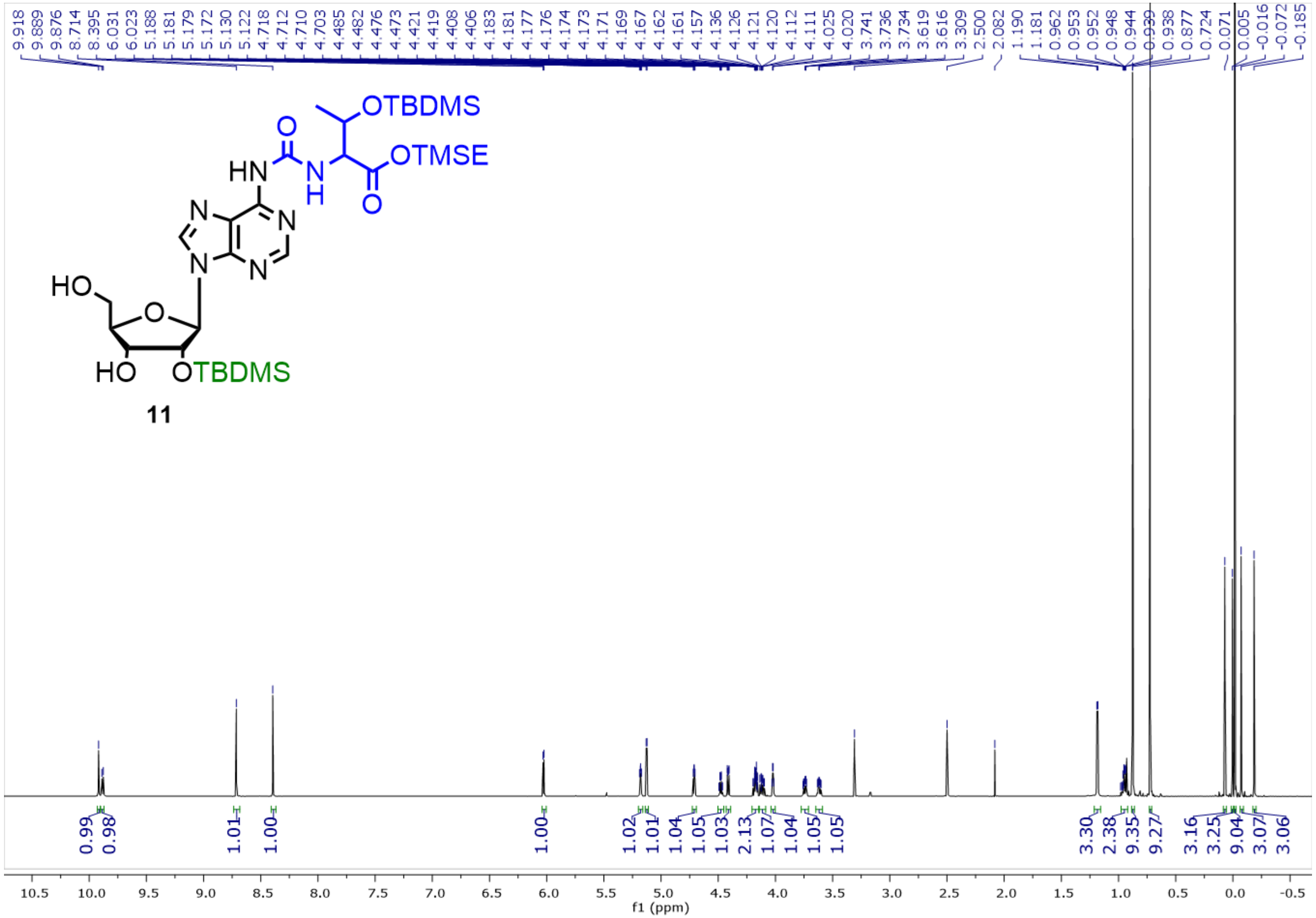

Figure S23 ^{13}C NMR spectrum of **11**

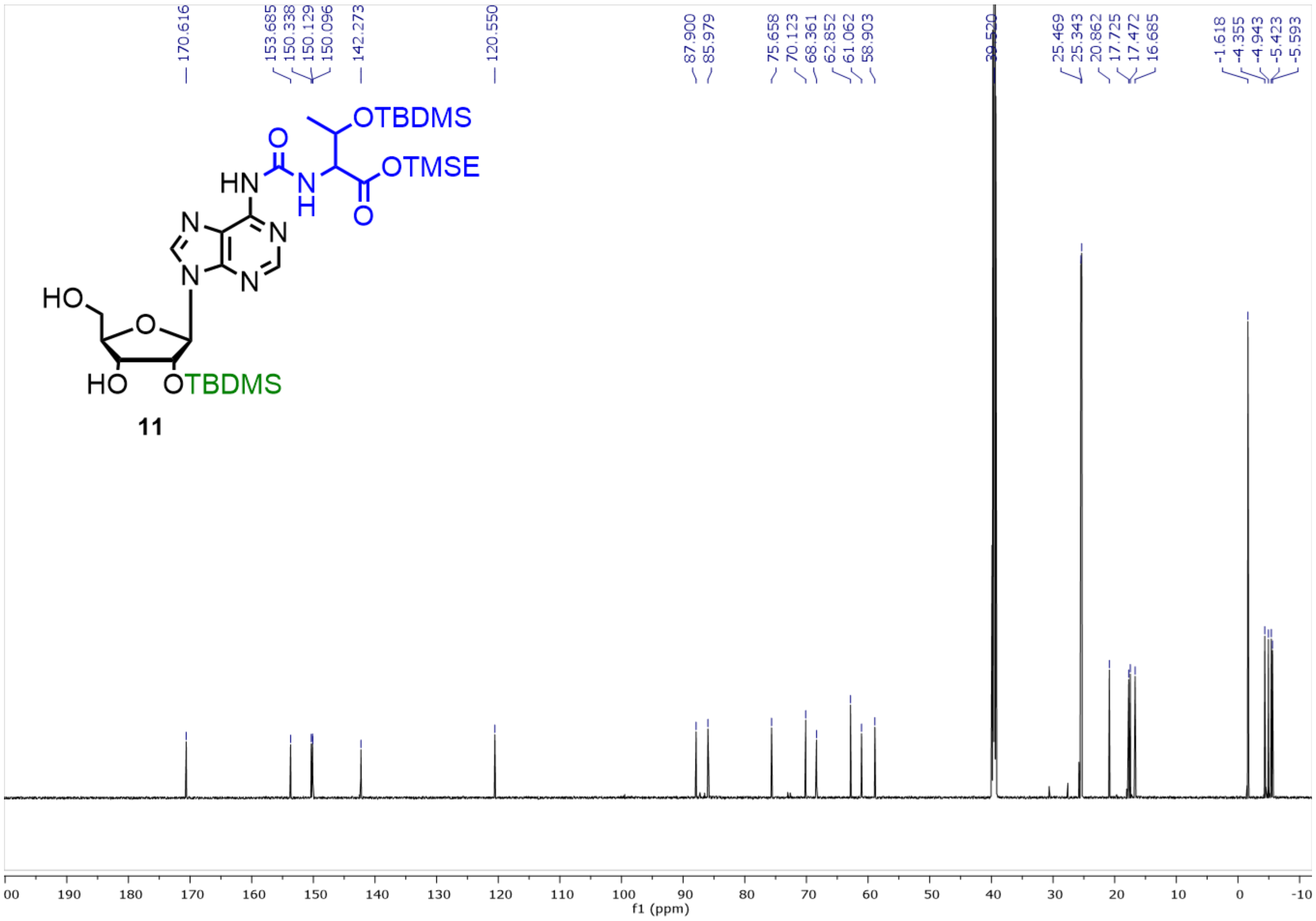

Figure S24 ^{1}H NMR spectrum of **12**

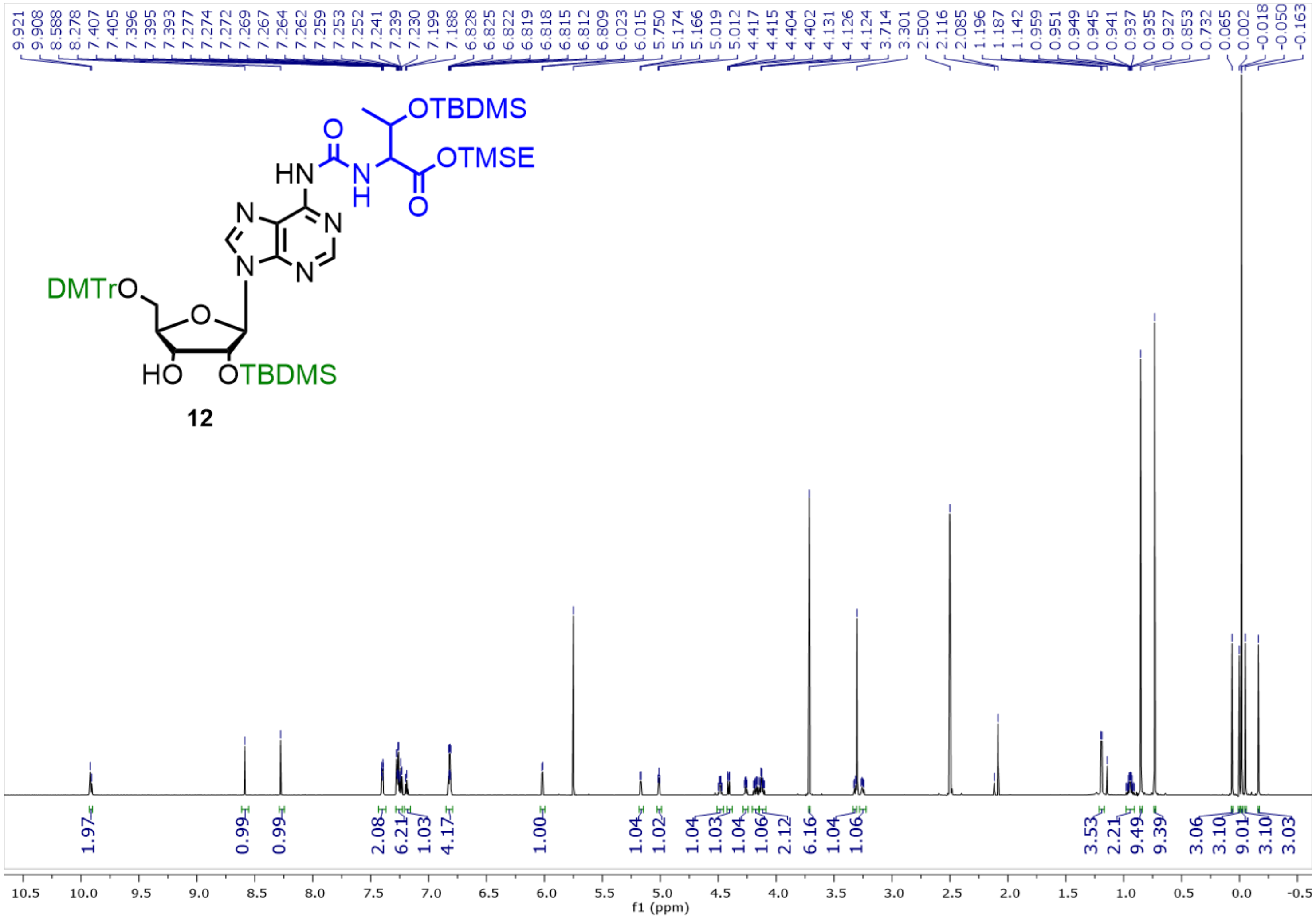

Figure S25 ^{13}C NMR spectrum of **12**

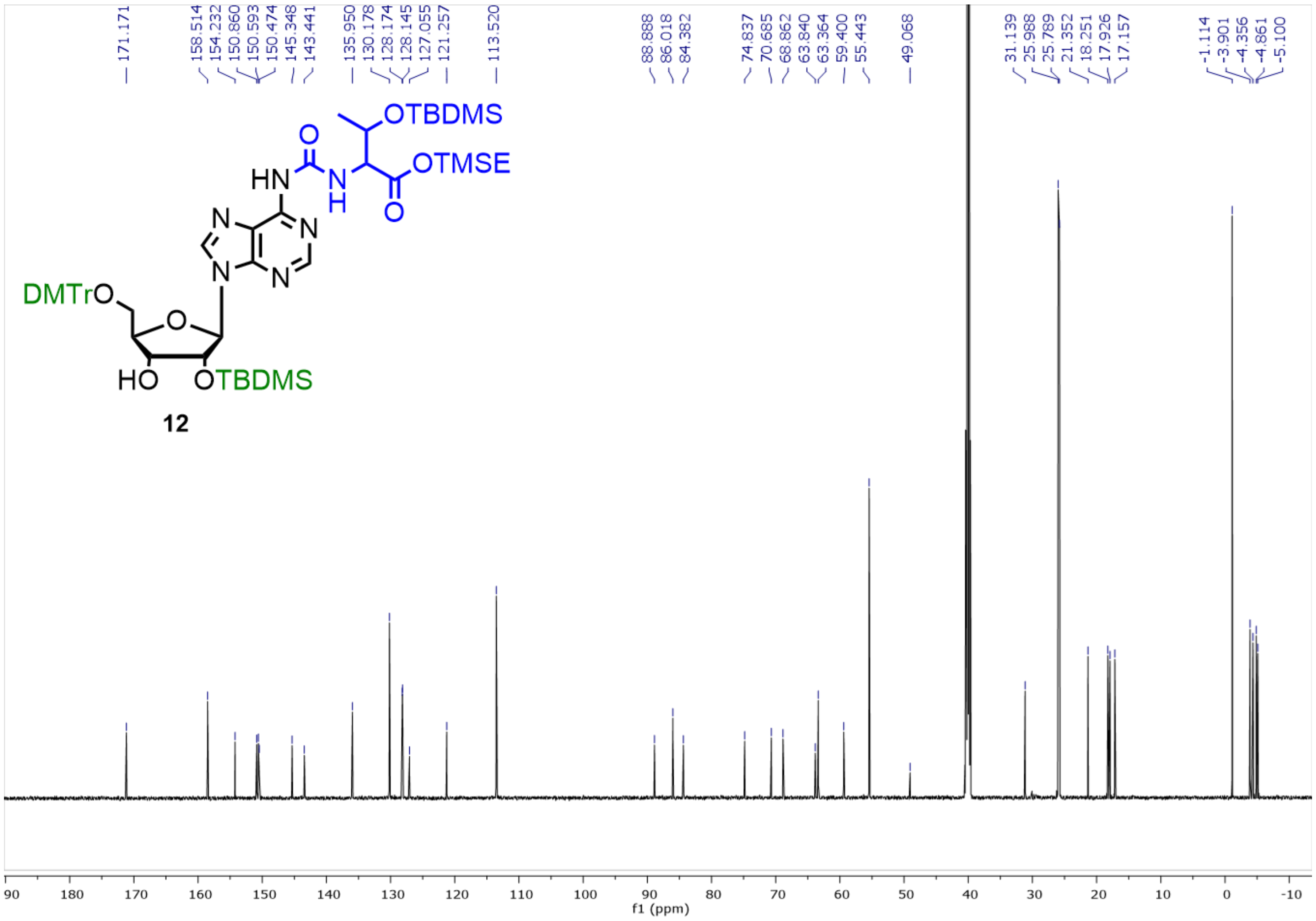

3.3 HR-MS spectra of adenosine derivatives.

Figure S26 ESI HR-MS spectrum of **4a**

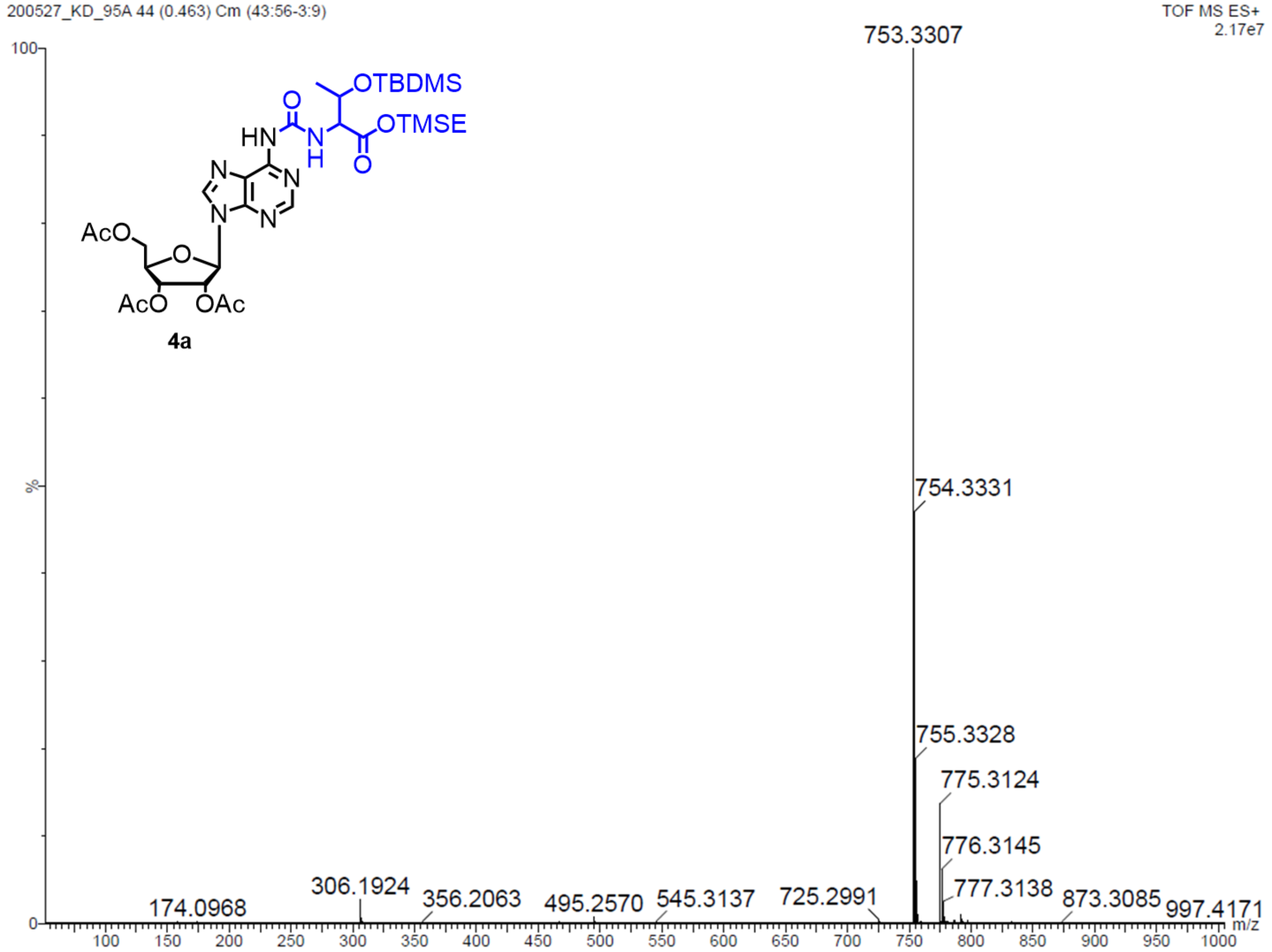

Figure S27 ESI HR-MS spectrum of **4b**

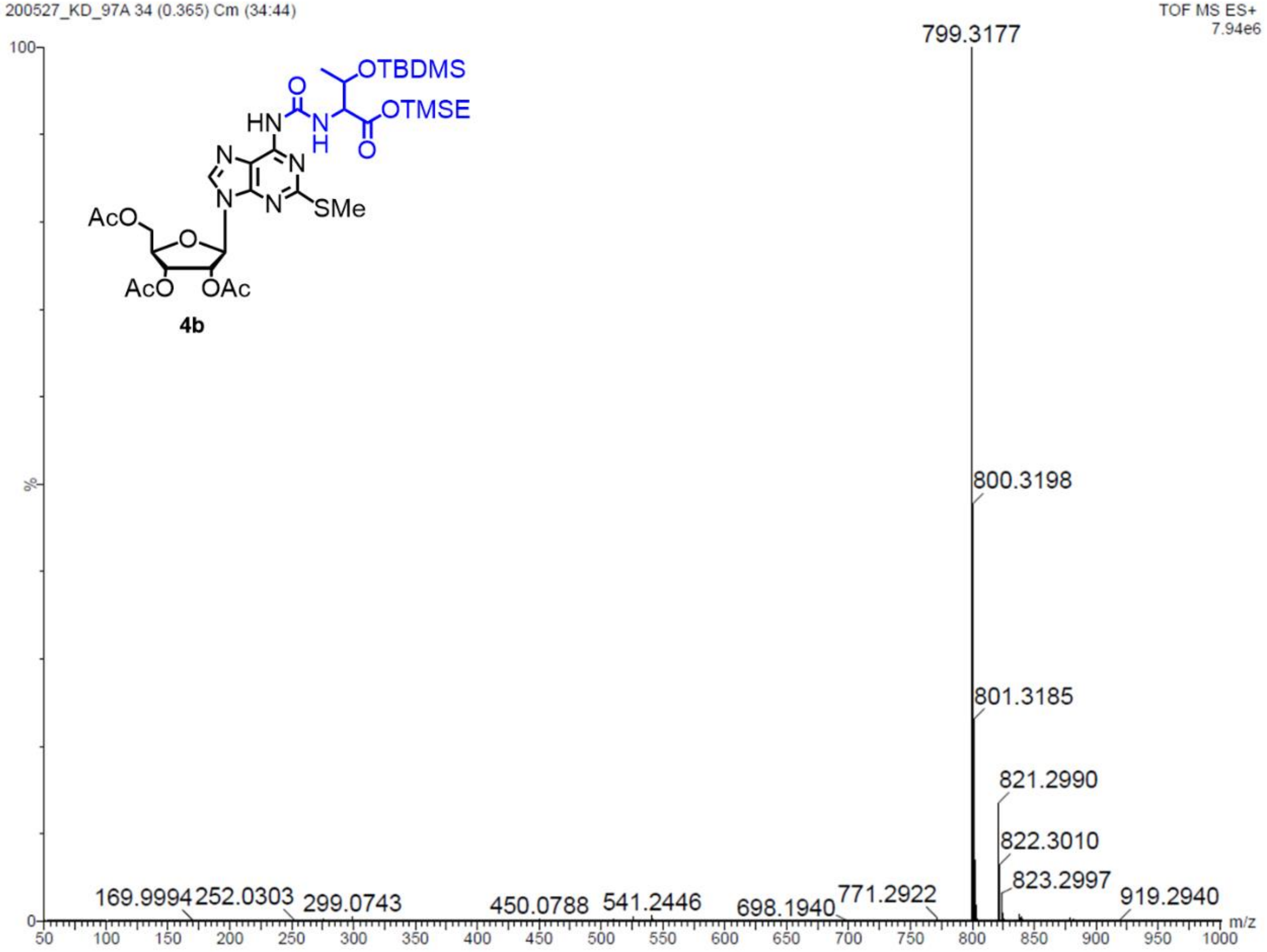

Figure S28 ESI HR-MS spectrum of **10**

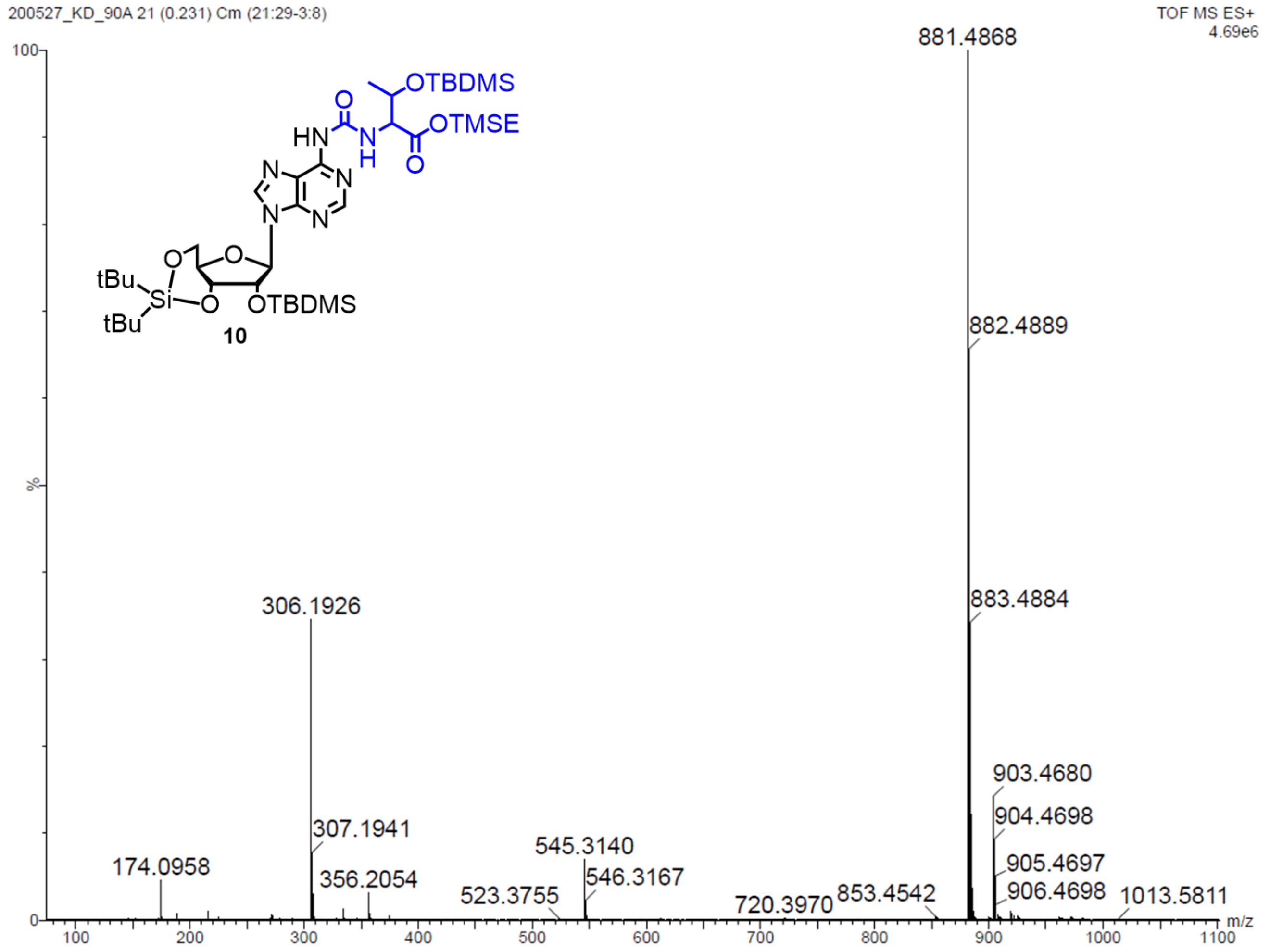

Figure S29 ESI HR-MS spectrum of **11**

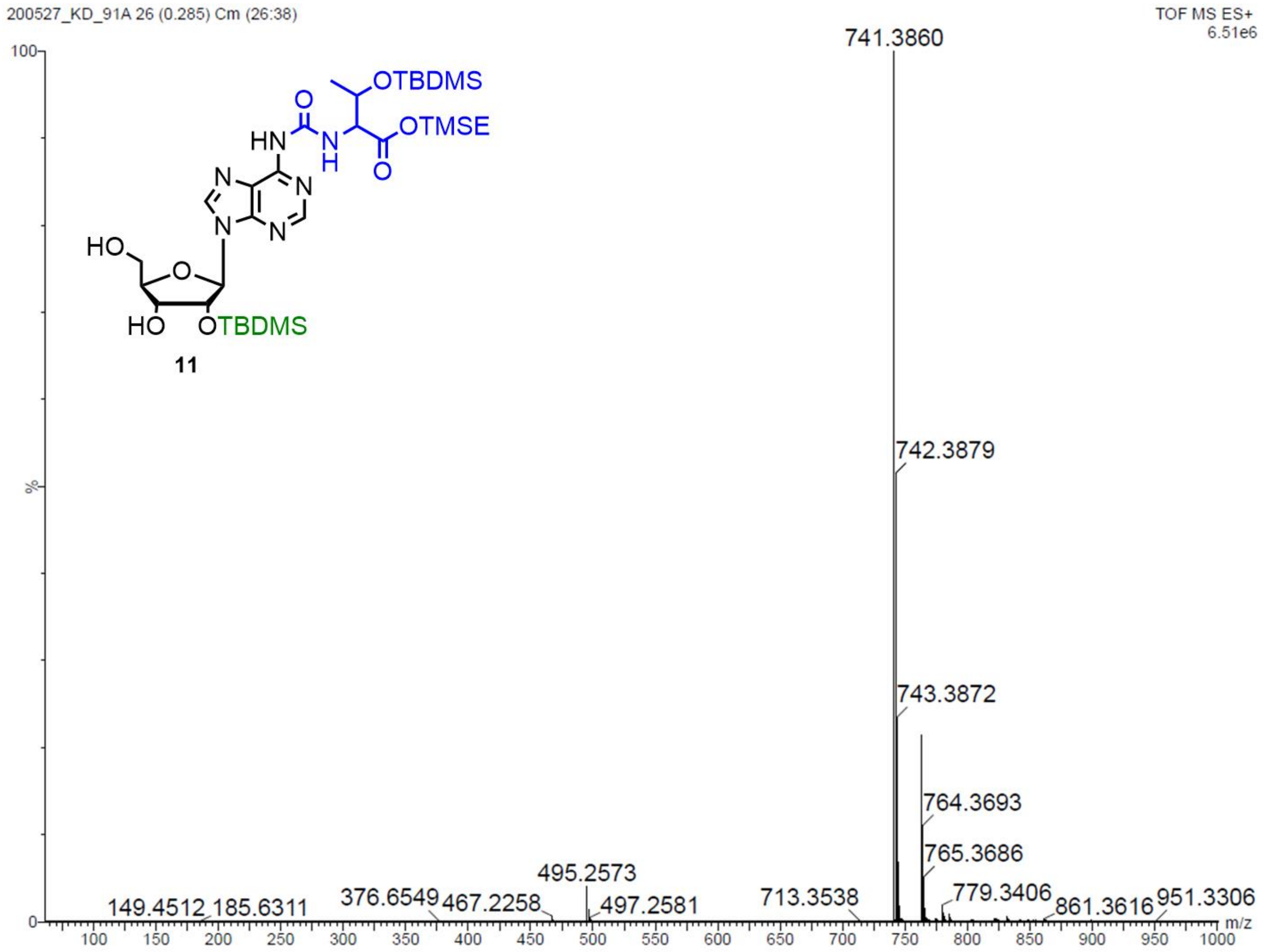

Figure S30 ESI HR-MS spectrum of **12**

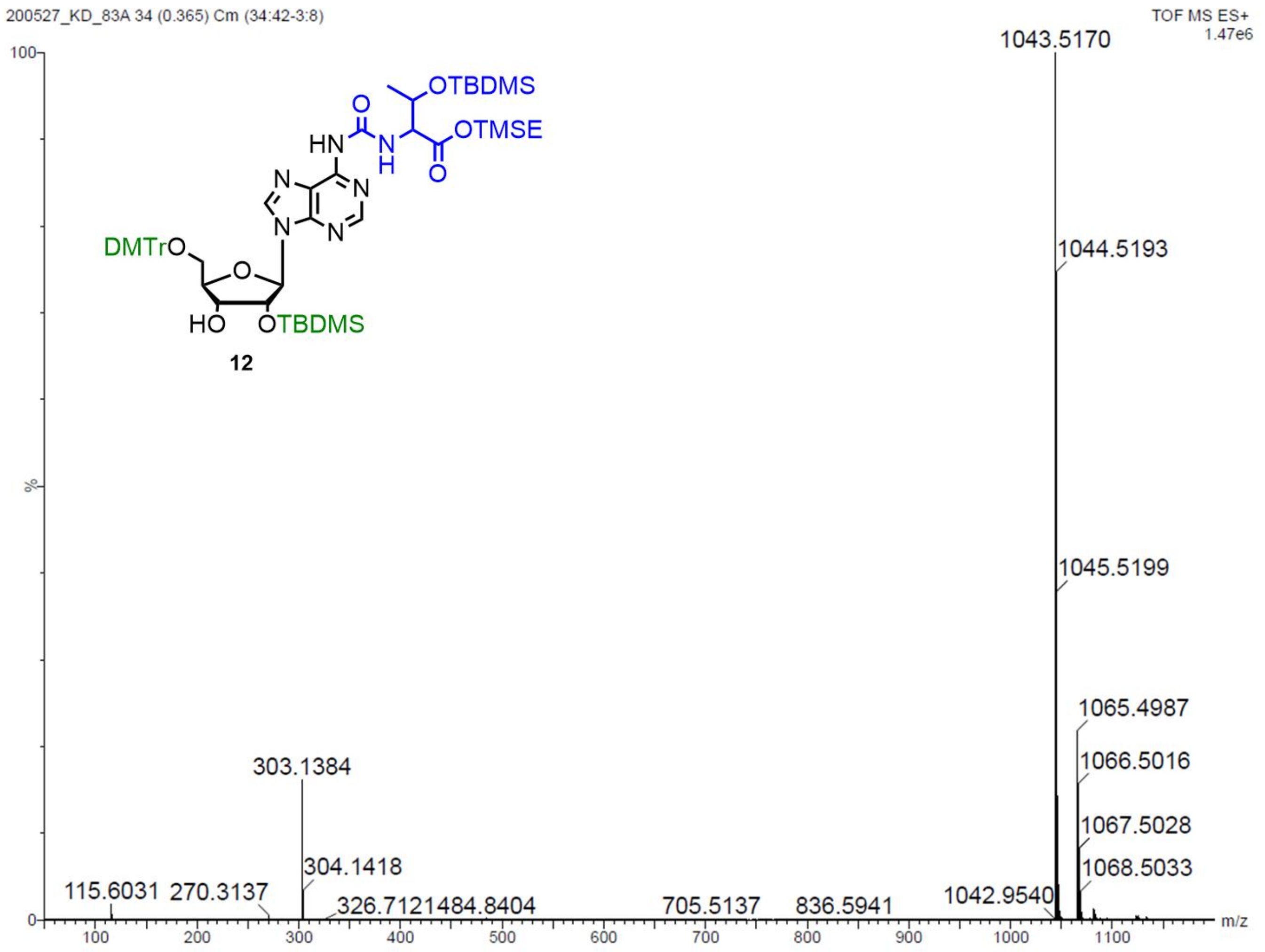

Supplement: RA-011-D0RA09803E-s001 [file RA-011-D0RA09803E-s001.pdf]
